# CNT‐Assembled Octahedron Carbon‐Encapsulated Cu_3_P/Cu Heterostructure by In Situ MOF‐Derived Engineering for Superior Lithium Storage: Investigations by Experimental Implementation and First‐Principles Calculation

**DOI:** 10.1002/advs.202000736

**Published:** 2020-05-29

**Authors:** Jia Lin, Chenghui Zeng, Xiaoming Lin, Chao Xu, Cheng‐Yong Su

**Affiliations:** ^1^ Key Laboratory of Theoretical Chemistry of Environment, Ministry of Education, Guangzhou Key Laboratory of Materials for Energy Conversion and Storage, School of Chemistry South China Normal University Guangzhou 510006 P. R. China; ^2^ College of Chemistry and Chemical Engineering, Key Laboratory of Functional Small Organic Molecule, Ministry of Education and Jiangxi's Key Laboratory of Green Chemistry Jiangxi Normal University Nanchang 330022 P. R. China; ^3^ MOE Laboratory of Bioinorganic and Synthetic Chemistry, Lehn Institute of Functional Materials, School of Chemistry Sun Yat‐Sen University Guangzhou 510275 P. R. China

**Keywords:** carbon nanotube‐assembled octahedra, copper phosphides, heterostructured anodes, lithium storage, metal–organic frameworks (MOFs)

## Abstract

Conspicuously, metal–organic frameworks (MOFs) serve as homogenously and periodically atom‐dispersed self‐sacrificial template for in situ engineering of hierarchical porous carbon‐encapsulated micro/nanoheterostructure materials, integrating the merits of micro/nanostructure to high‐volumetric energy storage. Copper phosphide represents a promising candidate due to its compact material density compared to commercial graphite. Herein, micro/nanostructured Cu_3_P/Cu encapsulated by carbon‐nanotube‐assembled hierarchical octahedral carbonaceous matrix (Cu_3_P/Cu@CNHO) is constructed by an in situ MOF‐derived engineering for novel anode material in LIBs, which achieves an extraordinary cycling stability (a well‐maintained gravimetric/volumetric capacity of 463.2 mAh g^−1^/1878.4 mAh cm^−3^ at 1 A g^−1^ up to 1600 cycles) and distinguished rate capability (an ameliorated capacity of 317.7 mAh g^−1^ even at 10 A g^−1^), together with unprecedented heat‐resistant capability (an elevated temperature of 50 °C for 1000 cycles maintaining 434.7 mAh g^−1^ at 0.5 A g^−1^). The superior electrochemical performance of Cu_3_P/Cu@CNHO is credited to the large specific surface area, conductive carbon matrix and metallic copper dopants, synergistic effects of the intrinsic Cu_3_P/Cu heterostructure, and well‐defined micro/nanostructure, facilitating a boosted electrochemical conductivity and accelerated diffusion kinetics.

## Graphical abstract

A facile in situ metal–organic framework‐derived engineering method is proposed for carbon‐nanotube‐assembled octahedron carbon‐encapsulated Cu_3_P/Cu heterostructure toward superior lithium storage (cycling stability, rate performance, and heat‐resistant capability). The quantitative kinetics analysis, ex situ characterizations, and density functional theory calculations further manifest the reversible conversion mechanism and the boosted kinetics of this micro/nanostructured Cu_3_P/Cu@CNHO anode with distinguished cyclability and rate performance.

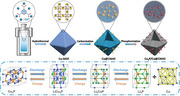

## 1 Introduction

Lithium‐ion batteries (LIBs), serving as a prevailing electrochemical energy storage and conversion technology, have exerted paramount significance for their applications in energy storage devices in virtue of high energy density and prolonged cycling performance.^[^ ^1^, ^2^ ^]^ Driven by the imperative demand for the superior energy density, exceptional rate capability, and safety concerns, graphite, as the state‐of‐the‐art commercialized anode material for LIBs, delivers limited energy density and low voltage plateau, triggering lithium dendrite formation at high rate. This impediment invigorates tremendous attentions to the promising anode alternatives to achieve higher volumetric capacity, distinguished cycling/rate durability, and safer voltage plateau.^[^ ^3^, ^4^ ^]^ On the one hand, elaborated nanostructure engineering has been proposed to considerably improve the rate capability and gravimetric specific capacity, but undesirably led to a low volumetric capacity with inherently inferior tap density.^[^ ^5^, ^6^, ^7^ ^]^ On the other hand, microstructure suffers from poor rate performance owing to intrinsic electrical and ionic insulation of transport, despite its high tap density. Consequently, constructing an optimized hierarchical micro/nanostructure by “bottom‐up” (encapsulating nanoparticles into micro‐bulk) or “top‐down” (constructing nanopores inside microstructure) engineering unfolds an exquisite strategy to accommodate the above hurdles.^[^ ^8^, ^9^, ^10^ ^]^ Additionally, it is necessitated to boost the electrochemical dynamics by furnishing an appropriate anode with large surface area, fast charge transfer, and improved electrochemical conductivity.^[^ ^11^, ^12^ ^]^

Recently, transition metal phosphides (TMPs), denoted as [M*_x_*P*_y_*] (where M = Zn, Ni, Fe, etc.), have been emerged as a research hotspot of LIB anode candidates via conversion reaction mechanism.^[^ ^13^, ^14^, ^15^ ^]^ Most of the TMPs (such as CoP, Ni_2_P, and FeP) achieve safe operating potential and high specific capacity due to their theoretical specific capacity. Unfortunately, their intrinsic tap density, inferior electronic conductivity, low porosity, and huge volumetric expansion pose a severe compromise on the electrochemical properties (including volumetric capacity and cycling durability) of TMPs for practical application.^[^ ^16^ ^]^ Nevertheless, copper phosphide (Cu_3_P, consisting of low‐cost and earth‐sufficient elements) is the competitive and promising one, considering its theoretical gravimetric capacity close to that of graphite (363 mAh g^−1^ for Cu_3_P and 372 mAh g^−1^ for graphite). Moreover, Cu_3_P achieves approximately three times higher volumetric capacity than that of graphite (3020 mAh cm^−3^ for Cu_3_P and 830 mAh cm^−3^ for graphite) stemming from its higher material density;^[^ ^17^ ^]^ meanwhile, it is evitable for Cu_3_P to the solvent co‐intercalation side reaction, but easily possible for graphite with gradually decreased specific capacity.^[^ ^18^, ^19^ ^]^ To mitigate these intractable puzzles, ingeniously hybrid superstructures are constructed by elaborate‐designed micro/nanostructure engineering aiming for enlarging the contact between electrode and electrolyte and offering more active sites upon lithiation/delithiation process.^[^ ^20^ ^]^ In addition, nanotube structures correlate with large surface area and inner channels, which are vital for electrons/ions and mass transfer in the electrodes.^[^ ^21^ ^]^ On the flip side, the hybridization of active materials with carbonaceous materials by embedding in a conductive carbon matrix (such as porous carbon shell, carbon nanotubes (CNTs), and carbon‐based materials), or anchoring on carbon substrate like graphene, not only boosts the electrochemical conductivity of the electrode and accelerates the electrochemical kinetics, but also alleviates the volume fluctuation and suppresses the aggregation.^[^ ^22^ ^]^ However, it is unsatisfactory for particles with their weak and insufficient interfaced reactions by simply anchoring on carbon substrate. Due to the dispersity preference of metal phosphides (in water) and conductive carbon substrate (in organic solvent), such electrode design suffers from material segregation between binder and active materials, which exhibits inferior cyclability and severe capacity fading.^[^ ^23^, ^24^ ^]^ Therefore, the ideal compacted Cu_3_P electrodes are anticipated to possess structural and compositional features on unique micro‐/nano‐ as well as carbon matrix encapsulated superstructure.

Metal–organic frameworks (MOFs), constructed by metal clusters and organic linkers, are a class of porous materials with their large specific surface area, appealing porosity, and controllable structures, which serve as self‐sacrificial templates to in situ engineering of porous carbon‐encapsulated TMPs@C electrode materials for LIBs in recent years.^[^ ^25^, ^26^ ^]^ Nevertheless, the TMPs with porous nanostructure can also buffer the strain, and the plentiful interior voids, facilitating the immersion of electrolyte in electrode materials and shorter ion diffusion pathways. Recent studies emphasize that TMPs distributed homogenously on the carbon matrix can further emerge as linkers to interconnect the individual particles for effective electrons/ions transfer, thus further improve the charge transfer kinetics and structural stability.^[^ ^27^ ^]^ Probably due to lacking of effective synthetic strategy, there are only two works on MOF‐derived Cu_3_P@C have been reported. For example, MOF‐derived Cu_3_P nanoparticles were designed for hydrogen evolution and oxygen reduction,^[^ ^28^ ^]^ and metal‐organophosphine framework (MOPF)‐derived Cu_3_P@C nanoparticles for sodium‐ion batteries.^[^ ^29^ ^]^ However, the expensive organophosphine ligand (such as PTA) and low synthetic yield made the synthesis of MOPF‐derived TMPs nanostructures relatively complicated and difficult to practical applications. Hence, achieving the design of TMPs@C micro/nanostructure by MOF‐derived strategy remains full of challenges. To the best of our knowledge, there are no reports on Cu‐MOF‐derived Cu_3_P/Cu encapsulated by in situ CNT‐assembled carbon matrix with a micro/nanostructural integrity as high volumetric capacity electrode for LIBs.

Motivated by the above‐mentioned considerations, herein, a micro/nanostructured Cu_3_P/Cu heterostructure encapsulated by CNT‐assembled hierarchical octahedral carbonaceous matrix (Cu_3_P/Cu@CNHO) is synthesized by MOF‐derived engineering as an exceptionally novel anode for LIBs. The in situ CNT‐assembled carbon matrix and structural integrity contribute to dispersing the binder and active materials during slurry preparation and inherit advantages for high volumetric capacity electrode; meanwhile, metallic Cu dopants boost the conductivity with synergistic effect by the ternary compositional features. The Cu_3_P/Cu@CNHO achieves unprecedented stability for long‐term cyclability (463.2 mAh g^−1^/1878.4 mAh cm^−3^ at 1 A g^−1^ up to 1600 cycles), rate capability (317.7 mAh g^−1^ even at 10 A g^−1^), and high‐temperature performance (434.7 mAh g^−1^ at 0.5 A g^−1^ after 1000 cycles), manifesting extraordinary electrochemical performance as anode for LIBs. In‐depth investigations systematically unravel the fabulous structural stability and the lithium storage mechanism of Cu_3_P/Cu@CNHO heterostructure. Furthermore, density functional theory (DFT) calculations simultaneously demonstrate that the elaborate‐designed architecture is furnished with strong intrinsic electronic interaction within the matrix, boosted electronic conductivity, low diffusion energy barriers, and superior rate performance, ultimately contributing to much faster lithium‐ion storage kinetics of Cu_3_P/Cu@CNHO than the Cu_3_P and Cu_3_P@CNHO. This work may shed promising lights on the MOFs‐derived engineering of micro/nanoheterostructure embedded in conductive carbon matrix anodes with ultralong cycle life and ultrahigh rate performance for LIBs.

## 2 Results and Discussion

### 2.1 Structure and Morphology Characterization

**Scheme 1:**
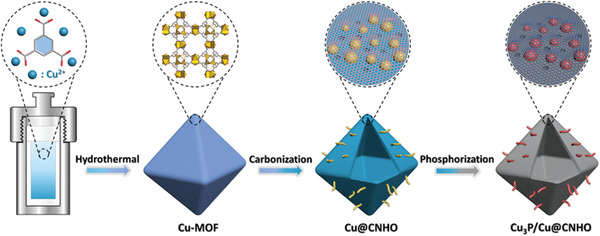
Schematic illustration for the synthetic strategy of CNT‐assembled micro/nanostructured Cu_3_P/Cu@CNHO heterostructure.

**Figure 1:**
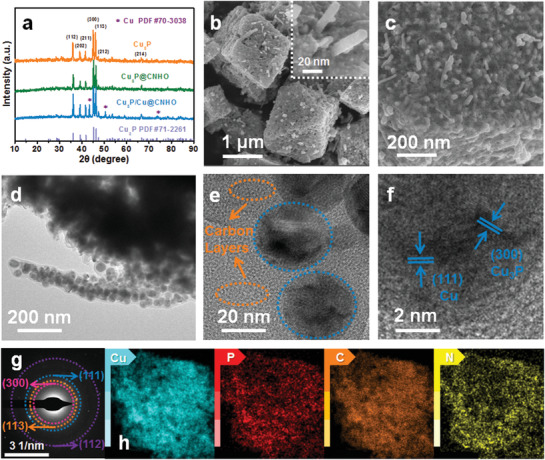
a) XRD patterns of the Cu_3_P, Cu_3_P@CNHO, and Cu_3_P/Cu@CNHO. b,c) SEM images of Cu_3_P/Cu@CNHO. d,e) TEM image of the particle fringe and the assembled CNT of Cu_3_P/Cu@CNHO. f) HRTEM images of Cu_3_P/Cu@CNHO. g) SAED pattern, and h) EDS elemental mapping of Cu, P, C, and N for Cu_3_P/Cu@CNHO.

The synthesis process of the CNT‐assembled micro/nanostructured Cu_3_P/Cu@CNHO is schematically depicted in **Scheme** **1**, achieved by a unique in situ MOF‐derived engineering. Briefly, the octahedron Cu‐MOF was first self‐assembled by Cu^2+^ and H_3_BTC ligand via facile solvothermal method. The field‐emission scanning electron microscope (FESEM) image of the Cu‐MOF exhibits octahedron morphology (Figure S1, Supporting Information). Powder X‐ray diffraction (PXRD) pattern and thermogravimetric analysis (TGA) unambiguously confirm the high phase purity and thermal stability of the as‐synthesized Cu‐MOF (Figures S2 and S3, Supporting Information). Subsequently, the Cu‐MOF was carbonized in nitrogen atmosphere to construct a CNT‐assembled hierarchical porous octahedron carbon‐encapsulated Cu hybrid (Cu@CNHO), which was further observed by XRD and SEM techniques. Interestingly, the carbonization treatment in N_2_ flow is advantageous to preserve the morphology of the Cu‐MOF, and the generated carbon during the first annealing might function as buffer frameworks to avoid severe structural contraction of MOFs for further phosphorization. As shown in Figures S4 and S5a in the Supporting Information, the diffraction peaks at 43.2°, 50.3°, and 73.9° of Cu@CNHO can be indexed to the (111), (200), and (220) planes of cubic metallic Cu (PDF#70‐3038), and the morphology of octahedron Cu@CNHO is assembled by self‐generated CNTs. After phosphidation of the Cu@CNHO with NaH_2_PO_2_ in argon flow, Cu@CNHO is controllably converted into Cu_3_P/Cu@CNHO at 250 °C, while pure Cu_3_P@CNHO can be ideally achieved at 300 °C, indicating the complete conversion of Cu component into Cu_3_P by increasing the phosphidated temperature. The characteristic diffraction peaks of the PXRD pattern in **Figure** **1**a can be well‐indexed to the standard hexagonal Cu_3_P (PDF#71‐2261) and cubic Cu (PDF#70‐3038) phase in Cu_3_P/Cu@CNHO. The corresponding crystal configuration details are described in Figure S6 in the Supporting Information. The Cu_3_P/Cu@CNHO inherits the shape characteristics of Cu@CNHO, showing a hierarchical porous octahedron morphology anchored with self‐generated CNTs with a relatively rough surface (Figure 1b and Figure S5b,d, Supporting Information). Following the different phosphidation treatment, the shape of Cu_3_P@CNHO is still furnished with a similar porous framework as Cu_3_P/Cu@CNHO (Figure S5c, Supporting Information). It is possible to observe that plenty of nanoparticles are elaborately dispersed on the micro‐octahedron, further constructing a conspicuous micro/nanostructure (Figure 1c). Additionally, during the carbonization and phosphidation processes, the released gases enable the copper ions to move outside, resulting in the formation of porous structures. The morphology of Cu_3_P/Cu@CNHO was further characterized by transmission electron microscopy (TEM), elucidating the hierarchical porous octahedrons are furnished with carbon nanotubes (Figure 1d). As presented in Figure 1e, the micro‐octahedron structure is assembled by considerable nanoparticles which are encapsulated in the carbon matrix, constructing the desirable micro/nanostructure. The high‐resolution TEM image of the Cu_3_P/Cu@CNHO reveals the lattice fringe spacings with interplanar distances of 0.201 and 0.209 nm covered by an amorphous carbon layer (Figure 1f), corresponding to the (300) crystal plane of Cu_3_P and (111) lattice facet of Cu, respectively, which is in accordance with the XRD results and demonstrates the coexistence of Cu_3_P and Cu in the products. According to Figure 1g, the corresponding selected area electron diffraction (SAED) pattern indicates that the major diffraction rings matched well with the (300), (113), and (112) facets of Cu_3_P and (111) plane of Cu in Cu_3_P/Cu@CNHO, implying the crystal configuration information. The energy‐dispersive X‐ray spectroscopy (EDS) elemental mapping images in Figure 1h reveal the homogenous element distributions of Cu, P, C, and N, suggesting the successful construction of Cu_3_P/Cu@CNHO. Elemental analysis indicates the carbon/nitrogen content to be 9.61/0.95 and 10.28/1.04 wt% for Cu_3_P@CNHO and Cu_3_P/Cu@CNHO, respectively. In addition, noncarbon sole Cu_3_P sample is obtained by the analogous phosphidation synthesis (Figure 1a).

**Figure 2:**
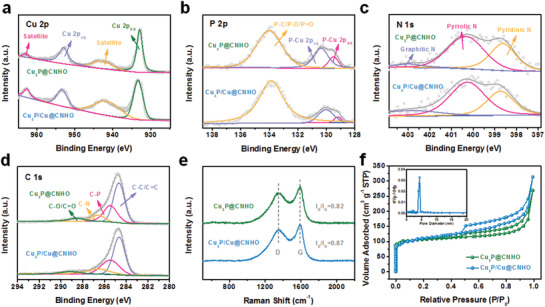
High‐resolution XPS spectra of a) Cu 2p, b) P 2p, c) N 1s, and d) C 1s in the Cu_3_P@CNHO and Cu_3_P/Cu@CNHO samples, respectively. e) Raman spectra and f) nitrogen adsorption–desorption curves of Cu_3_P@CNHO and Cu_3_P/Cu@CNHO.

To investigate the bonding states and chemical compositions, Cu_3_P, Cu_3_P@CNHO, and Cu_3_P/Cu@CNHO were characterized by X‐ray photoelectron spectroscopy (XPS). The XPS survey‐scan spectrum reveals the coexistence of Cu, P, C, O, and N elements in the as‐prepared samples (Figure S7, Supporting Information). The Cu 2p core‐level spectra can be deconvoluted into four subpeaks for Cu_3_P@CNHO and Cu_3_P/Cu@CNHO, respectively (**Figure** **2**a), with characteristic subpeaks (932.9 and 953.0 eV for Cu_3_P@CNHO, 933.3 and 953.5 eV for Cu_3_P/Cu@CNHO) corresponding to Cu 2p_3/2_ and Cu 2p_1/2_ of Cu─P, respectively.^[^ ^30^ ^]^ Moreover, two more satellite peaks indicate the shakeup excitation of high‐spin Cu ions. In addition, the P 2p spectrum can be deconvoluted into three peaks at 129.2, 130.1, and 133.9 eV (Figure 2b). The peaks at 129.2 and 130.1 eV in the P 2p core level are indexed to P‐Cu 2p_3/2_ and P‐Cu 2p_1/2_ in Cu_3_P, along with the peaks at 133.9 eV related to the P─C, P─O, and P═O, respectively.^[^ ^12^, ^30^ ^]^ The subpeaks in the N 1s spectrum are ascribed to nitrogen species including pyridinic nitrogen (398.8 eV), pyrrolic nitrogen (400.3 eV), and graphitic nitrogen (403.1 eV) (Figure 2c), where these nitrogen species are beneficial for promoting the discharge capacity due to the furnished electron defects.^[^ ^28^, ^31^ ^]^ As illustrated in Figure 2d, the high‐resolution C 1s spectra are well fitted by four peaks for graphitic carbon (C═C/C─C), carbon bonding with phosphorus, oxygen, and nitrogen (C─P, C─O, and C─N).^[^ ^32^, ^33^ ^]^ Moreover, Raman spectroscopy of Cu_3_P@CNHO and Cu_3_P/Cu@CNHO is depicted in Figure 2e to study the amorphous structure character of the as‐synthesized samples. Two representative bands of carbon matrices at 1345 and 1587 cm^−1^ are designated to the disordered sp^3^ carbon (D‐band) and graphitic sp^2^ carbon (G‐band), respectively. The intensity ratios *I*_D_/*I*_G_ for the Cu_3_P@CNHO and Cu_3_P/Cu@CNHO heterostructures are 0.82 and 0.87, respectively, demonstrating the high graphitization degree of carbon matrices in these materials.^[^ ^34^, ^35^, ^36^ ^]^ The carbon matrices can not only effectively boost the electronic conductivity, but also unambiguously accommodate the exfoliation of Cu_3_P due to the intractable volume changes during intercalation/deintercalation processes.^[^ ^34^ ^]^

Ascribing to the high porosity of MOF precursor and the emitted gases during carbonization and phosphidation progress, N_2_ absorption–desorption isotherms at 77 K in Figure 2f indicate that the Cu_3_P@CNHO and Cu_3_P/Cu@CNHO possess hysteresis loops of the typical type IV adsorption/desorption isotherm leading to the high porosity. The Brunauer–Emmett–Teller surface area takes up to 262.7 and 350.4 m^2^ g^−1^ for Cu_3_P@CNHO and Cu_3_P/Cu@CNHO, respectively. The pore size distribution mainly centers at 2 and 4 nm, calculated by the Barrett–Joyner–Halenda method (Figure 2f, inset and Figure S8, Supporting Information). The large specific surface area and the unique nanoporous structure can afford shorter diffusion paths and more active sites, guarantee expedited electrolyte access to inner positions, and can be beneficial for facilitating lithium‐ion transfer, further boosting the electrochemical performance of the heterostructure. Furthermore, Cu_3_P/Cu@CNHO possesses a considerably higher tap density (4.06 g cm^−3^) than that of commercial graphite anode.^[^ ^37^ ^]^ Figure S9 in the Supporting Information visually exhibits the volume of Cu_3_P/Cu@CNHO and commercial graphite anode materials with the same weight, indicating the higher mass loading of active materials in Cu_3_P/Cu@CNHO anode than commercial graphite with the same electrode‐coating thickness, and hence contributing toward a higher volumetric capacity.

### 2.2 Electrochemical Properties

**Figure 3:**
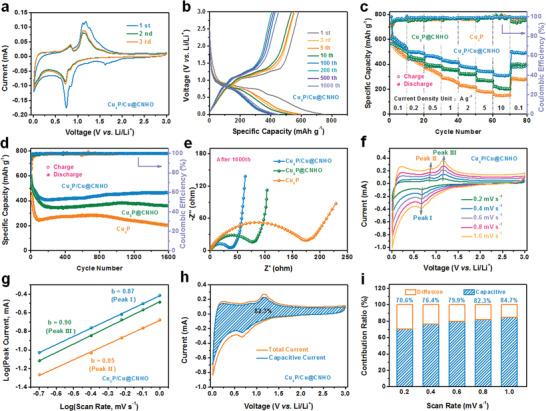
a) CV curves of the Cu_3_P/Cu@CNHO heterostructured electrodes at the scan rate of 0.2 mV s^−1^ with a voltage window from 0.01 to 3.0 V. b) The galvanostatic charge/discharge profiles of Cu_3_P/Cu@CNHO electrodes at 1 A g^−1^. c) Rate capabilities at various current densities ranging from 0.1 to 10 A g^−1^ of the Cu_3_P, Cu_3_P@CNHO, and Cu_3_P/Cu@CNHO electrodes, respectively. d) Long‐term cyclabilities at 1 A g^−1^, and e) Nyquist plots after 1600th cycle of Cu_3_P, Cu_3_P@CNHO, and Cu_3_P/Cu@CNHO electrodes. Kinetics analysis of the Li‐ion storage performance and quantitative analysis for the pseudocapacitive contribution for Cu_3_P/Cu@CNHO anode. f) CV plots at increasing sweep rates from 0.2 to 1.0 mV s^−1^. g) Calculations for the determinational *b* values of main cathodic and anodic peaks. h) Separation of the pseudocapacitive and diffusion‐controlled contribution by CV curves at 0.8 mV s^−1^. i) Contribution percentages of the pseudocapacitive‐controlled contributions at corresponding sweep rates.

To gain further insights into this MOF‐derived CNT‐assembled octahedron Cu_3_P/Cu@CNHO heterostructure, the electrode films were assembled by CR2032 half‐cells with Li metal as the counter electrode. For comparison, the initial three cyclic voltammetry (CV) profiles at a sweep rate of 0.2 mV s^−1^ for Cu_3_P, Cu_3_P@CNHO, and Cu_3_P/Cu@CNHO electrodes are recorded in **Figure** **3**a and Figure S10 in the Supporting Information to study their electrochemical mechanisms. Accordingly, CV profiles of all the three electrodes are similar to each other along with the characteristic lithiation/delithiation peaks of Cu_3_P electrodes. In the first cathodic scan in Figure 3a, an extra reduction peak at around 1.62 V is ascribed to the decomposition of the electrolyte, and side reaction of CuO stemming from the long‐time exposure of Cu_3_P into air and then the slight oxidation into copper oxides.^[^ ^16^ ^]^ The distinct peaks near 0.85 and 0.74 V correlate with the conversion reaction from Cu_3_P into Li*_x_*Cu_3−_ *_x_*P and metallic Cu, and the one below 0.17 V corresponds to the generation of the Li_3_P and Cu. In the first anodic process, the oxidation peaks presented at around 0.84, 1.15, and 1.31 V can be assigned to reversible delithiation and phase transitions of Li_3−_ *_x_*Cu*_x_*P,^[^ ^16^, ^38^, ^39^ ^]^ which is further confirmed by this work. More impressively, no additional oxidation peak appears for the oxidation of Cu into CuO above 2.5 V, indicating a common but negligible content of CuO in the as‐synthesized Cu_3_P materials, which is in line with the reported Cu_3_P materials. The CV curves of Cu_3_P/Cu@CNHO overlap in the following CV scans, endowing the stable conversion reversibility.

The galvanostatic charge/discharge (GCD) measurements were conducted to evaluate the electrochemical performance of the as‐prepared Cu_3_P/Cu@CNHO electrodes. Figure 3b depicts the charge/discharge curves of the Cu_3_P/Cu@CNHO at different cycling depth at a current density of 1 A g^−1^. The long voltage plateaus at about 0.86 and 0.73 V are attributed to the conversion reactions of Cu_3_P, which is identical to the CV results. The Cu_3_P/Cu@CNHO achieves the initial discharge/charge capacity of 727.3/558.8 mAh g^−1^ with a related initial Coulombic efficiency (CE) of 76.8%, which the irreversible capacity originates from the generation of the solid electrolyte interface (SEI) film, decomposition of electrolyte, and incomplete decomposition of Li_3_P. By contrast, the initial discharge capacities for Cu_3_P and Cu_3_P@CNHO electrodes exhibit 749.7 mAh g^−1^ (74.7%) and 750.5 mAh g^−1^ (75.3%) (Figure S11, Supporting Information), respectively. Significantly, although all the three samples deliver similar initial discharge capacities, the Cu_3_P undergoes severe capacity attenuation in the following prolonged cycles (Figure S11b, Supporting Information), showing a comparatively inferior cyclability of the lithium storage performance for Cu_3_P.

Rendering as a prominent parameter for evaluating the high‐power storage performance of anodes, the rate capabilities of the Cu_3_P, Cu_3_P@CNHO, and Cu_3_P/Cu@CNHO electrodes are depicted in Figure 3c under gradually increased current densities. Seemingly, the rate capability of Cu_3_P/Cu@CNHO achieves higher capacity than that of both Cu_3_P and Cu_3_P@CNHO. The discharge capacities of 499.1, 460.8, 418.2, 381.9, and 345.3 mAh g^−1^ can be observed with the increasing current densities of 0.2, 0.5, 1, 2, and 5 A g^−1^, respectively, which exhibits a slightly decreasing trend. Even at a harsh current density of 10 A g^−1^, Cu_3_P/Cu@CNHO still shows a distinguished discharge capacity of 317.7 mAh g^−1^, whereas the Cu_3_P and Cu_3_P@CNHO go through dramatic degradation of electrochemical performance with the increasing current density, endowing the superior rate capability of Cu_3_P/Cu@CNHO than Cu_3_P and Cu_3_P@CNHO. Moreover, the capacity of Cu_3_P/Cu@CNHO is well‐maintained when the current density recovers back to 0.1 A g^−1^, which is anticipated to be the functions of conductive Cu metal nanocore in interior structure of the material and the protective carbon matrices on boosting the electron transfer and alleviating the volume fluctuation during lithiation/delithiation process. To further investigate the advantages of such a CNT‐assembled Cu_3_P/Cu@CNHO heterostructure with conductive Cu nanocore and carbon matrix, the long‐term cycling stability at a high current density of 1 A g^−1^ for Cu_3_P, Cu_3_P@CNHO, and Cu_3_P/Cu@CNHO anodes is presented in Figure 3d, which the volumetric specific capacities have been evaluated by the calculation methods described in the Supporting Information and Figure S12 (Supporting Information). It can be observed that Cu_3_P and Cu_3_P@CNHO gradually exhibit a degenerated cycling capacity as the cycle number increased and hence deliver poorer cyclic stability, which may be ascribed to the inferior electronic conductivity, poor structural stability, and deficient synergistic effect of the composition. Cu_3_P/Cu@CNHO retains an exceptionally gravimetric/volumetric capacity of 463.2 mAh g^−1^/1878.4 mAh cm^−3^ at 1 A g^−1^ up to 1600 cycles, which is superior to those of Cu_3_P (202.7 mAh g^−1^) and Cu_3_P@CNHO (359.2 mAh g^−1^). Nevertheless, the CE of Cu_3_P/Cu@CNHO increases rapidly after the second cycle and behaves ≈100% due to the dwindling of the generation of SEI and decomposition of electrolyte, manifesting the cycling stability and reversibility of Cu_3_P/Cu@CNHO electrode.

The heat‐resistant capability of LIBs is associated with the safety and stability concerns, which predominantly depends on the structure and composition of the electrode materials.^[^ ^40^ ^]^ Figure S13 in the Supporting Information depicts the cyclability of Cu_3_P/Cu@CNHO at 0.5 A g^−1^ at an elevated temperature of 50 °C. The Cu_3_P/Cu@CNHO maintains almost 434.7 mAh g^−1^ of gravimetric capacity after 1000 cycles. The initial CE of Cu_3_P/Cu@CNHO is 78.3%, which recovers almost 100% with the following cycles. Hence, the structural and compositional advantages of sophisticated micro/nanostructure engineering for Cu_3_P/Cu@CNHO are transparent at the above elevated temperature, suggesting practical prospective application of the Cu_3_P/Cu@CNHO electrode under harsh conditions.

To reveal the mechanism for the excellent Li‐ion storage capability of Cu_3_P/Cu@CNHO, kinetics of the Cu_3_P, Cu_3_P@CNHO, and Cu_3_P/Cu@CNHO electrodes are investigated via electrochemical impedance spectroscopy (EIS) measurements after the initial deep cycle with the complete formation of a fresh SEI film, as well as after 1600 cycles (Figure 3e and Figure S14, Supporting Information). The semicircle from the high to intermediate frequency is equivalent to the charge transfer resistance (*R*_ct_) and the intercept of the semicircle is correlative to the electronic resistance (*R*_s_). The slope line in low‐frequency region coincides with the Warburg factor (*σ*) related to the lithium‐ion diffusion. The Nyquist plots show that the semicircle and slope of the straight line of Cu_3_P/Cu@CNHO are smaller than that of Cu_3_P and Cu_3_P@CNHO, manifesting the electronically conductive Cu nanocore dopants and carbon matrix can expedite the lithium‐ion diffusion. More importantly, both the smaller semicircle and increased slope of the straight line of Cu_3_P/Cu@CNHO after 1600 cycles verify the gradual activation of this hybrid electrode with more boosted kinetics than that of the fresh cell, accompanied by the opposite behaviors of Cu_3_P.

As for the lithiation process, Li^+^ transfers from the Li foil toward the as‐prepared electrode contributing to different lithium storage mechanisms: a) masses of Li^+^ adsorb around the interface of the carbon matrix and negative electrons theoretically appear on the other side of carbon matrix, which further constructs a typical electric double layer capacitor for exceptional specific capacity (denoted as capacitive storage mechanism);^[^ ^41^, ^42^ ^]^ b) another part of Li^+^ diffuses through the carbon matrix and electrochemically reacts with the Cu_3_P active material (termed as diffusion‐controlled mechanism). To further interpret the electrochemical kinetics of the Cu_3_P/Cu@CNHO heterostructure for its distinguished cycling stability and rate capability, the CV measurements at scan rates from 0.2 to 1.0 mV s^−1^ were investigated on the Cu_3_P, Cu_3_P@CNHO, and Cu_3_P/Cu@CNHO electrodes. As illustrated in Figure 3f and Figures S15a and S16a in the Supporting Information, the redox peak currents of Cu_3_P/Cu@CNHO, Cu_3_P@CNHO, and Cu_3_P simultaneously upgrade with increasing sweep rates. Generally, the pseudocapacitive contributions can be determined by the power‐law relationship of the peak current (*i*) and scan rate (*v*)^[^ ^43^ ^]^

(1) $$\begin{array}{c} i=av^{b} \end{array}$$

in which *a* and *b* are constant parameters. If the *b* value equals to 0.5, a total diffusion‐controlled behavior is dominant in the storage kinetics; whereas 1.0 represents an ideal capacitive‐controlled mechanism.^[^ ^29^ ^]^ For Cu_3_P/Cu@CNHO, the corresponding *b* values of the main redox peaks are calculated to be 0.87, 0.85, and 0.90 (Figure 3g), which are higher than Cu_3_P@CNHO (0.77, 0.68, and 0.69) and Cu_3_P (0.64, 0.68, and 0.63) (Figures S15b and S16b, Supporting Information), confirming that the Cu_3_P/Cu@CNHO and Cu_3_P@CNHO composites are qualified with preponderantly significant capacitive‐controlled electrochemical kinetics to that of Cu_3_P due to the existence of carbon matrix. The contribution from the diffusion‐controlled reactions and capacitive‐controlled processes can be quantitatively separated by the following formula^[^ ^44^ ^]^

(2) $$\begin{array}{c} i\left(V\right)=k_{1}\nu +k_{2}\nu^{1/2} \end{array}$$

where the *k*_1_ *ν* corresponds to the pseudocapacitive process, while *k*_2_ *ν* ^1/2^ refers to the diffusion‐controlled capacity. As depicted in Figure 3h, the capacitive process of Cu_3_P/Cu@CNHO contributes 82.3% to the total capacity at the sweep rate of 0.8 mV s^−1^, which is higher than that of Cu_3_P@CNHO (66.3%) and Cu_3_P (51.9%) (Figures S15c and S16c, Supporting Information). Meanwhile, the pseudocapacitance contribution increases with the increasing sweep rates, where the pseudocapacitive fraction of Cu_3_P/Cu@CNHO and Cu_3_P@CNHO is higher than that of Cu_3_P (Figure 3i and Figures S15d and S16d, Supporting Information), implying that assembled‐CNT and embedded carbon matrix provide numerous lithium‐ion sites to store on the surface of material in virtue of the capacitive mechanism, considerably suppressing electrode material from destruction and further correlating with the improved cycling stability. Progressively, shorter ion transport pathway as well as boosted electron transfer can be realized by the conductive doped‐Cu formation and the high porosity, thereby facilitating the superior rate capability of Cu_3_P/Cu@CNHO heterostructure. Rendering as promising high energy density anode, the Cu_3_P/Cu@CNHO has a superior volumetric capacity in the cycle performance than the recently proposed representative transition metal oxide‐based and C‐based LIB anodes (Figure S17, Supporting Information). Additionally, the cyclic and rate capability of Cu_3_P/Cu@CNHO in contrast with other Cu_3_P anode electrodes for LIBs reported in recent years are depicted in Table S1 in the Supporting Information, which endows the superior electrochemical performance of Cu_3_P/Cu@CNHO material over the proposed representative Cu_3_P LIB anodes in gravimetric capacity.

**Figure 4:**
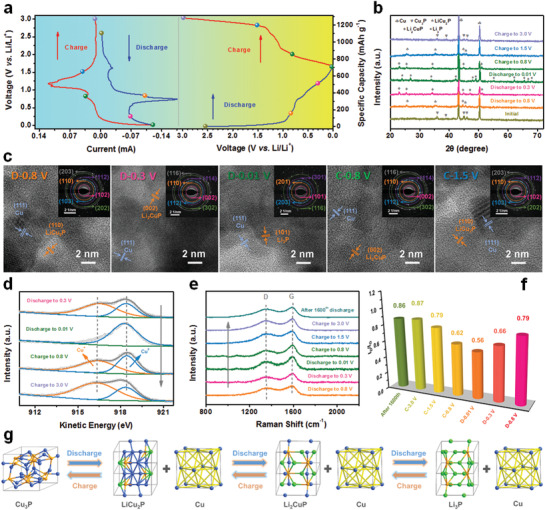
Electrochemical mechanisms of Cu_3_P/Cu@CNHO for LIBs. a) CV profile (left) and galvanostatic charge/discharge curves (right) with the marked cycled states. b) Ex situ XRD patterns and c) ex situ HRTEM images and corresponding SAED patterns of the selected electrodes’ discharged/charged stages. d) Ex situ L_3_M_45_M_45_ XAES spectra at the marked cycled states. e) Ex situ Raman spectra and f) corresponding *I*_D_/*I*_G_ values of Cu_3_P/Cu@CNHO at different intercalation/deintercalation states. g) Schematic illustrations of electrochemical mechanism of Cu_3_P during the charge/discharge processes.

To elucidate a comprehensive concept of the lithium‐ion storage mechanism of Cu_3_P/Cu@CNHO electrodes, ex situ analysis techniques including XRD patterns, HRTEM characterizations, SAED patterns, XPS profiles, and Raman spectra are depicted in **Figure** **4**. Unambiguously, Figure 4a illustrates the different states of the lithiation/delithiation process for kinetics analysis. As shown in Figure 4b, the corresponding diffraction peaks of Cu_3_P and Cu are investigated by the XRD pattern of the pristine electrode material, whereas the strong peaks of Cu can be somewhat ascribed to the Cu foil substrate and the doped‐Cu in the Cu_3_P/Cu@CNHO heterostructure. As the initial lithiation to 0.8 and 0.3 V, the diffraction intensity of the Cu_3_P characteristic peaks gradually fades out, while the weak diffraction peaks of LiCu_2_P (PDF#25‐0481) and Li_2_CuP (PDF#25‐0479) arise, confirming the lithium intercalation into Cu_3_P lattice interstices, and the formation of intermediate phase (Li*_x_*Cu_3−_ *_x_*P). Upon further lithiation to 0.01 V, it can be observed that the peaks of Li_2_CuP leisurely disappear, but the peaks correlating with the (101) lattice facet of Li_3_P (PDF#04‐0525) appear. Upon following delithiation process, the diffraction peaks of Li_2_CuP are observed at around 0.8 V, but almost disappeared when delithiating back to 1.5 and 3.0 V. The LiCu_2_P phase appears on delithiation to 1.5 V, and disappears after fully delithiating, illuminating that the conversion reactions mainly occur between 0.8 and 1.5 V during delithiation process. The aforementioned mechanism demonstrates a typical conversion reaction, which is directly confirmed by the HRTEM images and SAED patterns of the sole electrodes without copper foil current collector (Figure 4c). Upon lithiation to 0.8 V and delithiation to 1.5 V, the lattice fringes and diffraction patterns of LiCu_2_P can be simultaneously observed, which the interlayer spacing of 0.20 nm is assigned to the (110) planes of LiCu_2_P. Meanwhile, the Li_2_CuP formation is detected by the corresponding lattice fringes and diffraction patterns, when the cell discharges to 0.3 V and charges to 0.8 V. Additionally, the interlayer spacing of 0.33 nm can be indexed to the (101) planes of Li_3_P after fully discharging to 0.01 V. During the delithiation/lithiation process, the existence of copper auxiliary illustrates the conversion mechanism, implying the highly reversible electrochemical reactions. What is more, the XPS characterization was carried out to verify the reaction mechanism. Since the characteristic binding energy of Cu^+^ and Cu^0^ are somewhat superimposed together, the specific chemical valences of Cu are precisely characterized via L_3_M_45_M_45_ X‐ray‐excited Auger electron spectroscopy (XAES) spectra (Figure 4d). Accordingly, the broad and asymmetric Auger peaks of Cu_3_P/Cu@CNHO electrode cycled at different stages are deconvoluted into two symmetrical peaks with the kinetic energy of around 917.1 and 918.2 eV, correlating to the existence of Cu^+^ and Cu^0^, respectively.^[^ ^45^, ^46^, ^47^ ^]^ Thus, the characteristic peak of Cu^+^ decreases upon lithiation progress. Especially, when fully discharged to 0.01 V, the peak of Cu^+^ disappears, illustrating the transition of copper from Cu^+^ to Cu^0^. Moreover, the characteristic peak of Cu^+^ then increases as fully delithiating to 3.0 V, whereas the peak of Cu^0^ weakens because of the reversible mechanism, which corresponds to the descent and then rise of Cu valence. Intriguingly, the metallic copper peaks are detected throughout the whole progress, which is compatible for the boosted conductivity and outstanding electrochemical diffusion kinetics. Based on the above‐mentioned analysis, the in‐depth insights of the electrochemical mechanism are schematically illustrated in Figure 4g, and the charging/discharging reactions of the Cu_3_P/Cu@CNHO electrode can be clarified as follows

(3) $$\begin{array}{c} Cu_{3}P+Li^{+}+e^{-}↔\mathrm{LiC}u_{2}P+\mathrm{Cu} \end{array}$$

(4) $$\begin{array}{c} \mathrm{LiC}u_{2}P+Li^{+}+e^{-}↔Li_{2}\mathrm{CuP}+\mathrm{Cu} \end{array}$$

(5) $$\begin{array}{c} Li_{2}\mathrm{CuP}+Li^{+}+e^{-}↔Li_{3}P+\mathrm{Cu} \end{array}$$

It is vital to study the changes in the disorder/graphitic degrees for D/G band of the carbon matrix to probe the structure stability and graphitization degree during the Li‐ion storage process, which is further associated with long‐term cyclability and high‐rate capacity.^[^ ^48^ ^]^ Herein, the Cu_3_P/Cu@CNHO electrode without the conductive additive (Super P) was prepared for Raman analysis (Figure 4e). Consequently, during the lithiation process, the *I*_D_/*I*_G_ value decreases from 0.79 to 0.56 when the voltage goes down from 0.8 to 0.01 V (Figure 4f), manifesting the increase in the graphitization degrees together with the insertion of lithium ions. Subsequently, the *I*_D_/*I*_G_ values exhibit an increasing trend from 0.62 to 0.87 during the delithiation process, endowing that the structure gradually recovers upon the delithiation. Meanwhile, the *I*_D_/*I*_G_ value of Cu_3_P/Cu@CNHO electrode after fully charging 1600 cycles was maintained at 0.86, indicating the eased volume expansion during the prolonged intercalation/deintercalation processes. The results can be further confirmed by the cross‐sectional SEM image of Cu_3_P/Cu@CNHO electrode after cycling (Figure S18, Supporting Information), in which the thickness of the electrode film was still well maintained at 13 µm.

### 2.3 DFT Calculations

**Figure 5:**
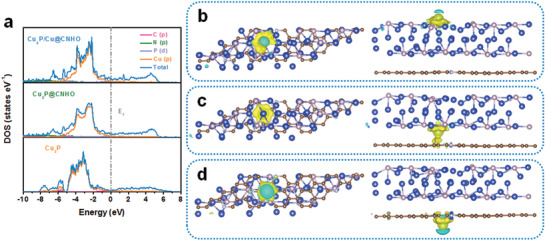
a) Total and orbital‐resolved partial DOS plots of Cu_3_P bulk, Cu_3_P@CNHO, and Cu_3_P/Cu@CNHO heterostructure. The Fermi levels (*E*_f_) are set to be 0 eV. Top and side views of differential charge density distribution of the Cu_3_P/Cu@CNHO heterostructure of Li b) adsorbing on the outer surface of Cu_3_P/Cu hybrid; c) inserting into the intralayer of Cu_3_P/Cu@CNHO; d) adsorbing on the outer surface of CNHO encapsulated‐carbon. Here, the accumulation of electrons is depicted in yellow regions, along with the depletion of electrons is shown in aquamarine regions.

DFT calculations were conducted to get more insights about the heterostructured interaction and distinguished electrochemical performance of the micro/nanostructured Cu_3_P/Cu@CNHO heterostructure. Herein, the structures of Cu_3_P bulk, Cu_3_P@CNHO, and Cu_3_P/Cu@CNHO are optimized and depicted in Figures S19–S21 in the Supporting Information, respectively. The hybridization of CNHO carbon matrix and metallic Cu core is deemed to boost the electronic conductivity of the Cu_3_P/Cu@CNHO material, which the electronic properties correspond to the electrochemical cyclability and rate performance of anode material in LIBs.^[^ ^49^, ^50^ ^]^ Therefore, the electronic structure calculations by density of states (DOS) bound up with the Fermi level (*E*_f_) were computed to investigate the total and orbital‐resolved partial DOS of the Cu_3_P bulk, Cu_3_P@CNHO, and Cu_3_P/Cu@CNHO heterostructure (**Figure** **5**a). The band structures of Cu_3_P render as a semiconductor with a band‐gap near the Fermi level. However, metallicities of the heterostructured Cu_3_P@CNHO and Cu_3_P/Cu@CNHO are observed with the distinct and continuous Fermi levels across the conduction bands. Impressively, the DOS of Cu_3_P/Cu@CNHO around the Fermi level is much denser than that of Cu_3_P@CNHO, indicating that the Cu dopants contribute to the effective improvement of electronic conductivity. The aforementioned results endow that more active electrons of Cu_3_P/Cu@CNHO heterostructure are accessible with the combination of carbonaceous matrix and the metallic Cu core. The conduction bands of Cu_3_P bulk, Cu_3_P@CNHO, and Cu_3_P/Cu@CNHO are predominantly taken up by empty Cu 3d states, while the valence bands are mainly composed of P 3p, C 2p, and N 2p states. Therefore, Cu_3_P/Cu@CNHO heterostructure exerts a considerable improvement in electronic conductivity comparable to that of Cu_3_P and Cu_3_P@CNHO, further accounts for the boosted electronic conductivity and prominent electrochemical capability.

Acknowledgedly, this synergistic effect is associated with the charge variation in the whole structure of the heterostructure.^[^ ^51^, ^52^ ^]^ Moreover, Bader charge distribution was implemented to investigate the interaction among the Li insertion into the Cu_3_P/Cu@CNHO heterostructure. Unambiguously, the possible insertion sites of heterostructured Cu_3_P/Cu@CNHO can be classified into three modes: I) Li adsorption on the outer surface of CNHO carbon matrix (Cu_3_P/Cu@CNHO‐Li); II) Li insertion into the intralayer of Cu_3_P/Cu@CNHO (Cu_3_P/Cu‐Li‐CNHO); III) Li adsorption on the outer surface of Cu_3_P/Cu system (Li‐Cu_3_P/Cu@CNHO). As summarized in **Table** **1**, the adsorbed Li mostly interacts with the outer surface carbon layer (CNHO carbon matrix) as for Cu_3_P/Cu@CNHO‐Li insertion mode, which the charge of Li diverts into the neighboring CNHO to generate a strong ionic bond. Concerning the Li‐Cu_3_P/Cu@CNHO pattern, the charge distribution shows the analogous ionic bond features together with the charge transferring from Li to the adjacent Cu_3_P/Cu hybrid with an average charge transfer of 0.227. Regarding as Li‐Cu_3_P/Cu@CNHO model for Li inserting into the intralayer of the heterostructured Cu_3_P/Cu@CNHO, the charge of Li simultaneously transfers to the adjacent CNHO and Cu_3_P/Cu clusters, which is contributed to an ovonic ionic bond and is different from the mode of Li adsorption on the outer surface of the Cu_3_P/Cu@CNHO heterostructure. In addition, it is noticeable that the CNHO matrix obtains more charge from Li than that of Cu_3_P or Cu_3_P/Cu cluster, associated with the strong electronegative difference between Li and N; herein, the CNHO matrix is anticipated to contribute to the cycling stability.

**Table 1:** The Bader charge distribution of Cu (△*Q*_Cu_), P (△*Q*_P_), C (△*Q*_C_), N (△*Q*_N_), and Li (△*Q*_Li_) atoms for Li adsorption on Cu_3_P bulk, Cu_3_P@CNHO, and Cu_3_P/Cu@CNHO heterostructure, which “+” means the loss of electrons and “−” stands for the gain of electrons

	Li Site	△*Q*_Cu_	△*Q*_P_	△*Q*_C_	△*Q*_N_	△*Q*_Li_
Cu_3_P	Cu_3_P‐Li	−0.043	+0.103	–	–	+0.439
Cu_3_P@CNHO	Cu_3_P@CNHO‐Li	−0.061	+0.165	+0.089	−0.885	+0.779
	Cu_3_P‐Li‐CNHO	−0.055	+0.140	+0.111	−0.963	+0.233
	Li‐Cu_3_P@CNHO	−0.055	+0.122	+0.112	−0.935	+0.387
Cu_3_P/Cu@CNHO	Cu_3_P/Cu@CNHO‐Li	−0.027	+0.077	+0.081	−0.838	+0.729
	Cu_3_P/Cu‐Li‐CNHO	−0.021	+0.050	+0.098	−0.869	+0.200
	Li‐Cu_3_P/Cu@CNHO	−0.010	+0.008	+0.010	−0.868	+0.227

Consequently, the interactions between Li and the Cu_3_P bulk, Cu_3_P@CNHO, and Cu_3_P/Cu@CNHO heterostructure are further manifested by differential charge density distributions. As depicted in Figure 5b–d and Figures S22 and S23 in the Supporting Information, the charge predominantly transfers from Li to the adjacent Cu_3_P, Cu_3_P/Cu clusters, or CNHO carbon matrix when Li adsorbs on the outer surface modes, verifying the formation of strong ionic bond among CNHO, Cu_3_P, or Cu_3_P/Cu clusters. Meanwhile, the charge diverts from Li into the neighboring CNHO and Cu_3_P (Cu_3_P/Cu) clusters (Figure 5c and Figure S23b, Supporting Information), as Li inserts into the intralayer of the Cu_3_P @CNHO (Cu_3_P/Cu@CNHO), affirming the electronic configuration change for the peculiar Cu‐doped structure as well as the strong charge interaction by encapsulated Cu_3_P/Cu clusters in the CNHO and carbon matrix. The results are in accordance with the data of Bader charge distributions, further endowing the beneficial ionic interaction between Li and the Cu_3_P/Cu@CNHO heterostructure and achieving the boosted electronic conductivity for prolonged cyclability and extraordinary rate capability of such an anode.

**Figure 6:**
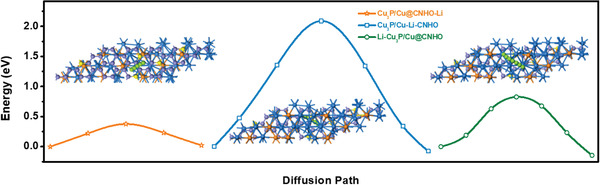
The diffusion pathways and corresponding calculated diffusion energy barrier profiles of Cu_3_P/Cu@CNHO heterostructure for Li migration on I) the outer surface of CNHO encapsulated‐carbon (Cu_3_P/Cu@CNHO‐Li), II) the intralayer of Cu_3_P/Cu@CNHO (Cu_3_P/Cu‐Li‐CNHO), and III) the outer surface of Cu_3_P/Cu hybrid (Li‐Cu_3_P/Cu@CNHO). The optimized diffusion paths are indicated by the small green spheres.

The mobility of Li on the anode decisively correlates with the rate capability of rechargeable LIBs.^[^ ^52^ ^]^ In order to investigate the lithium‐ion diffusion pathway and corresponding energy barriers in the asymmetry of Cu_3_P/Cu@CNHO heterostructure, we have also calculated the possible migration of Li based on three kinds of patterns as illustrated in **Figure** **6**: I) Li diffusion on the outer surface of CNHO carbon matrix (Cu_3_P/Cu@CNHO‐Li); II) Li diffusion in the intralayer of Cu_3_P/Cu@CNHO (Cu_3_P/Cu‐Li‐CNHO); III) Li diffusion on the outer surface of the Cu_3_P/Cu system (Li‐Cu_3_P/Cu@CNHO). Considering the pathways for lithium‐ion migration, the lowest diffusion energy barrier of 0.38 eV should be overcome when Li travels along Cu_3_P/Cu@CNHO‐Li path; nevertheless, the higher diffusion energy barriers of 2.09 and 0.83 eV for Cu_3_P/Cu‐Li‐CNHO and Li‐Cu_3_P/Cu@CNHO are observed, respectively (Figure 6), demonstrating that the diffusion path of Cu_3_P/Cu@CNHO heterostructure is dominated by Cu_3_P/Cu@CNHO‐Li pathway. Similarly, as for Cu_3_P@CNHO, Li moves along the Cu_3_P@CNHO‐Li pathway with a higher diffusion energy barrier of 0.41 eV, simultaneously surmounts the harsh energy barriers of 2.39 eV (Cu_3_P‐Li‐CNHO) and 1.12 eV (Li‐Cu_3_P@CNHO), as shown in Figure S24 in the Supporting Information. However, Li diffuses on the outer surface of Cu_3_P facilitating with a diffusion energy of 1.13 eV (Figure S25, Supporting Information). The above‐mentioned results anticipate that when the Li ions travel along Cu_3_P/Cu@CNHO, a considerably decreased energy barrier of 0.38 eV is achieved by the diffusion on Cu_3_P/Cu@CNHO‐Li and is dominant for the Li diffusion during the whole lithiation/delithiation process, compared with the higher value of 1.13 eV for monolayer Cu_3_P and 0.41 eV for Cu_3_P@CNHO, consequently demonstrating the boosted Li‐ion diffusion and intercalation kinetic of Cu_3_P/Cu@CNHO. All the migration barriers of diffusion pathways for the Cu_3_P/Cu@CNHO heterostructure are well‐optimized and decreased, illustrating the N‐doped CNHO‐encapsulated carbon matrix and metallic Cu core heterostructure have an intriguing charge/discharge capacity and superior rate performance for LIB. Henceforth, the migration path along Cu_3_P/Cu@CNHO‐Li with the lowest energy barriers provides the definitive advantages for fast lithium‐ion diffusion, and distinguished rate capability, together with the other higher diffusion energy pathways (Li‐Cu_3_P/Cu@CNHO and Cu_3_P/Cu‐Li‐CNHO) to construct a systematic connection of diffusion network of Cu_3_P/Cu@CNHO heterostructure, which may be mainly ascribed to the hybrid outer surface CNHO carbon matrix.

## 3 Conclusion

In summary, we proposed a novel synthetic strategy for in situ engineering of MOF‐derived micro/nanostructured Cu_3_P/Cu encapsulated by CNT‐assembled hierarchical octahedral carbonaceous matrix. Ascribing to the large specific surface area, conductive carbon matrix and metallic copper dopants, synergistic effects of the intrinsic Cu_3_P/Cu heterostructure, and the well‐assembled micro/nanostructure, the Cu_3_P/Cu@CNHO anode delivers prolonged cycling stability (463.2 mAh g^−1^ at 1 A g^−1^ after 1600 cycles), superior rate capability (317.7 mAh g^−1^ even at 10 A g^−1^), and unprecedented heat‐resistant capability (434.7 mAh g^−1^ at 0.5 A g^−1^ under 50 °C for 1000 cycles). The ex situ experimental and theoretical DFT characterizations were systematically conducted to get more insights into the lithiation/delithiation mechanism, confirming the diffusion and capacitance processes, reversible conversion mechanism, improved conductivity, low diffusion energy, and strong electronic interaction between carbon matrix and Cu_3_P/Cu heterostructure. We anticipate that this work gives new perspectives on in situ engineering of MOF‐derived carbon‐encapsulated metal phosphides/metal hybrids and the theoretical study of the relationship between the heterostructure and electrochemical mechanism for functional anode materials.

## 4 Experimental Section

##### Synthesis of Cu‐MOF

In a typical procedure, 0.72 g Cu(NO_3_)_2_, 0.44 g H_3_BTC, and 0.25 g polyvinylpyrrolidone were dispersed in 30 mL solvent of deionized (DI) water, ethanol, and dimethylformamide (DMF; 3:2:1, ratios by the volume) by ultrasonic dispersion for 30 min. Then, the mixture was sealed in to a 100 mL Teflon‐lined autoclave and reacted at 90 °C for 24 h. After naturally cooling down to ambient temperature, the blue powders were separated by centrifugation, and subsequently washed with DMF for at least three times followed by vacuum drying at 60 °C for 24 h.

##### Synthesis of CNT‐Assembled Cu_3_P@CNHO and Cu_3_P/Cu@CNHO Heterostructures

The as‐prepared Cu‐MOF was annealed at 600 °C for 2 h with a heating rate of 2 °C min^−1^ under a nitrogen flow. After naturally cooling to ambient temperature, CNT‐assembled carbon‐encapsulated Cu particles (Cu@CNHO) were obtained. To prepare the copper phosphide samples, 0.3 g of Cu@CNHO powders and 3.0 g of NaH_2_PO_2_·H_2_O as the P source were mixed for phosphorization. The mixtures were heated in the argon atmosphere with a heating rate of 10 °C min^−1^ at 250 and 300 °C, respectively. Finally, the as‐synthesized samples were collected by washing with DI water and ethanol for several times, and subsequently vacuum dried at 60 °C for 24 h. Finally, the as‐prepared products were denoted as Cu_3_P/Cu@CNHO (product for 250 °C) and Cu_3_P@CNHO (product for 300 °C), respectively.

##### Morphological and Structural Characterization

Bruker‐AXS D8 Advance system with a Cu K*α* radiation was conducted on measuring the crystal phase with PXRD in the 2*θ* range from 10° to 90°. Renishaw inVia confocal Raman microscope provision with an argon ion laser beam was implemented to test Raman spectra. Netzsch Thermo Microbalance TG 209 F1 Libra was employed to observe TGA from ambient temperature to 900 °C with a heating rate of 5 °C min^−1^ under nitrogen flow. Belsorp max gas sorption analyzer was used to analyze the sorption isotherms at 77 K. *K*‐Alpha^+^ XPS spectrometer (Thermo fisher Scientific, USA) was operated using Al K*α* radiation to evaluate XPS and the elemental compositions. Moreover, FESEM (TESCAN Maia 3, Czech) and TEM (FEI Talos F200X, USA) with high‐angle annular dark‐field (HAADF) STEM and EDS (JEM2010‐HR, 200 kV) were utilized to study the surface morphology and architecture.

##### Electrochemical Measurements

Electrochemical performances were measured on CR2032 coin‐type cells, in which Cu_3_P, Cu_3_P@CNHO, or Cu_3_P/Cu@CNHO was employed as working electrode and a Celgard 2400 membrane as separator, and 1 m LiPF_6_ was dissolved in an organic solvent mixture of ethylene carbonate and diethyl carbonate (EC:DEC:EMC, 1:1:1 by volume) as electrolyte, and the coin cell assembly was finished in the Ar‐filled glovebox (H_2_O ≤ 0.1 ppm, O_2_ ≤ 0.1 ppm). The electrodes were prepared by coating a slurry containing 90 wt% as‐synthesized active materials, 5 wt% acetylene black (Super P), and 5 wt% polyvinylidene fluoride dispersed in methylpyrrolidone on copper foil and vacuum drying at 100 °C for 24 h. Furthermore, Land battery tester (CT 2001A, Wuhan, China) was employed to perform GCD cycling tests between 0.01 and 3.0 V at 25 °C. The CV measurements at different scan rates and EIS tests were implemented on electrochemical workstation (CHI‐760E, Shanghai, China) with the frequency ranging from 100 kHz to 0.01 Hz and an amplitude of 5 mV.

##### Computational Methods

First‐principles calculations were carried out with the Vienna ab initio Simulation Package based on the DFT to investigate the distinguished electrochemical performance of the proposed heterostructures.^[^ ^53^ ^]^ The projector augmented wave method computed by the Kohn–Sham equations and the generalized gradient approximation method with the scheme of Perdew–Burke–Ernzerholf were adopted to study the electronic interaction of all the systems.^[^ ^54^ ^]^ The interlayer van der Waals (vdW) interactions were correlated with the Cu_3_P@CNHO and Cu_3_P/Cu@CNHO heterostructures for standard DFT description through the Grimme's D2 scheme.^[^ ^55^ ^]^ A thickness of 30 Å vacuum between the layers was optimized to eliminate the interaction of the periodically repeated boundary. In addition, the cutoff energy for plane‐wave expansion was considered to be 450 eV, and a Monkhorst–Pack^[^ ^56^ ^]^ k‐point mesh of 2 × 3 × 1 was constructed. As for the calculations, the convergence tolerances were chosen to be less than 10^−5 ^eV for the total energy difference, below 0.01 eV Å^−1^ for the final forces on all atoms. Charge details were computed through Bader analysis, containing the core charges, and charge density difference analysis within Vesta.^[^ ^57^ ^]^ The DOS was calculated via the Gaussian smearing method setting with a smearing width of 0.05 eV. To further investigate the diffusion pathways and energy barriers of Li in the Cu_3_P, Cu_3_P@CNHO, and Cu_3_P/Cu@CNHO heterostructure, the climbing image nudged elastic band method was used to illustrate the boosted electrochemical kinetics Cu_3_P/Cu@CNHO heterostructure as a superior anode for LIBs.^[^ ^58^, ^59^ ^]^

## Conflict of Interest

The authors declare no conflict of interest.

## Supporting information

Supporting Information
