# Supplementary material for: CNT‐Assembled Octahedron Carbon‐Encapsulated Cu3P/Cu Heterostructure by In Situ MOF‐Derived Engineering for Superior Lithium Storage: Investigations by Experimental Implementation and First‐Principles Calculation
Source: Adv Sci (Weinh). 2020 May 29;7(14):2000736. doi: 10.1002/advs.202000736 (PMC7375241; doi:10.1002/advs.202000736)
Supplement: Supplementary file 1 — Supporting Information [file ADVS-7-2000736-s001.pdf]

Electronic Supplementary Information (ESI)

CNT-Assembled Octahedron Carbon-Encapsulated Cu_3P/Cu Heterostructure by In-Situ MOF-Derived Engineering for Superior Lithium Storage: Investigations by Experimental Implementation and First-Principles Calculation

Jia Lin[a], *Chenghui Zeng*[b], *Xiaoming Lin*[a,*], *Chao Xu*[a,*], *Cheng-Yong Su*[c,*]

[a] Key Laboratory of Theoretical Chemistry of Environment, Ministry of Education, Guangzhou Key Laboratory of Materials for Energy Conversion and Storage, School of Chemistry, South China Normal University, Guangzhou 510006, China

[b] College of Chemistry and Chemical Engineering, Key Laboratory of Functional Small Organic Molecule, Ministry of Education and Jiangxi's Key Laboratory of Green Chemistry, Jiangxi Normal University, Nanchang 330022, China

[c] MOE Laboratory of Bioinorganic and Synthetic Chemistry, Lehn Institute of Functional Materials, School of Chemistry, Sun Yat-Sen University, Guangzhou 510275, China

AUTHOR INFORMATION

E-mail: linxm@scnu.edu.cn; chaoxu@m.scnu.edu.cn; cesscy@mail.sysu.edu.cn

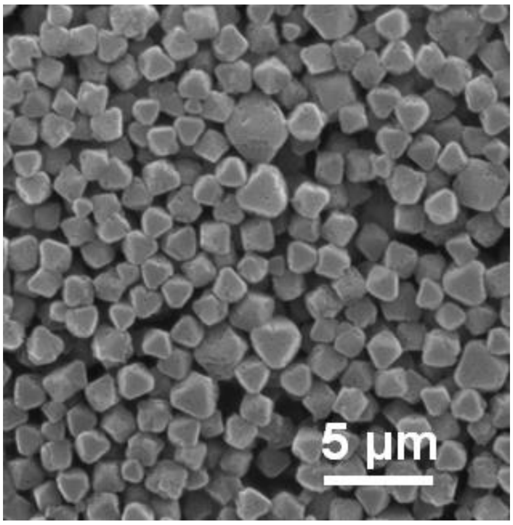

Figure S1. SEM image of Cu-MOF.

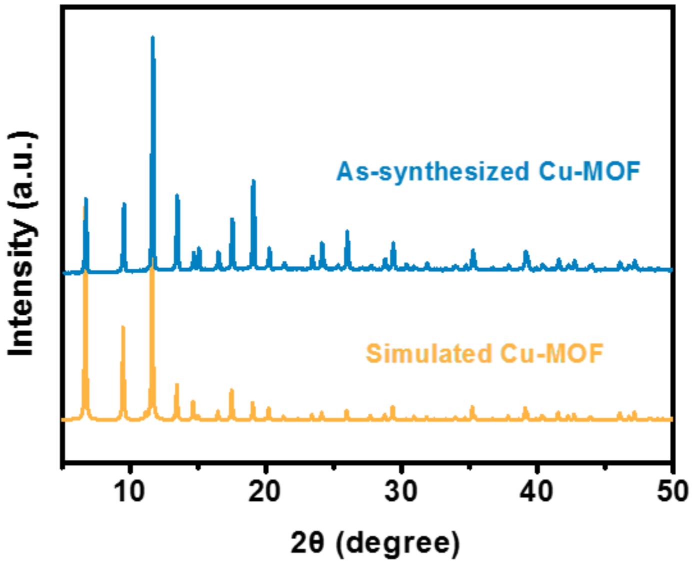

Figure S2. XRD pattern of as-synthesized Cu-MOF.

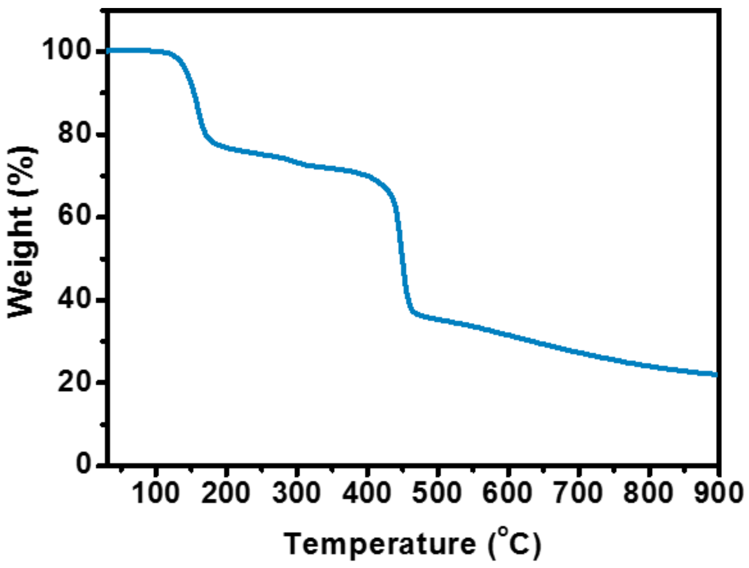

Figure S3. TGA plot of the Cu-MOF under nitrogen atmosphere.

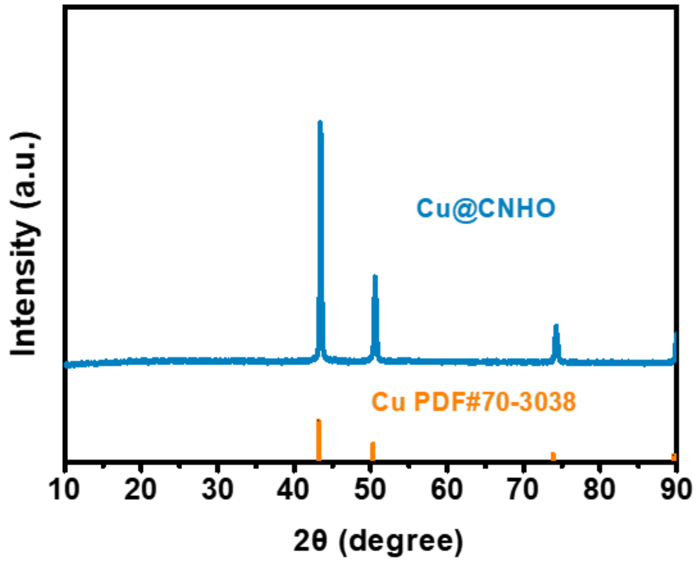

Figure S4. XRD pattern of Cu-MOF-derived Cu@CNHO.

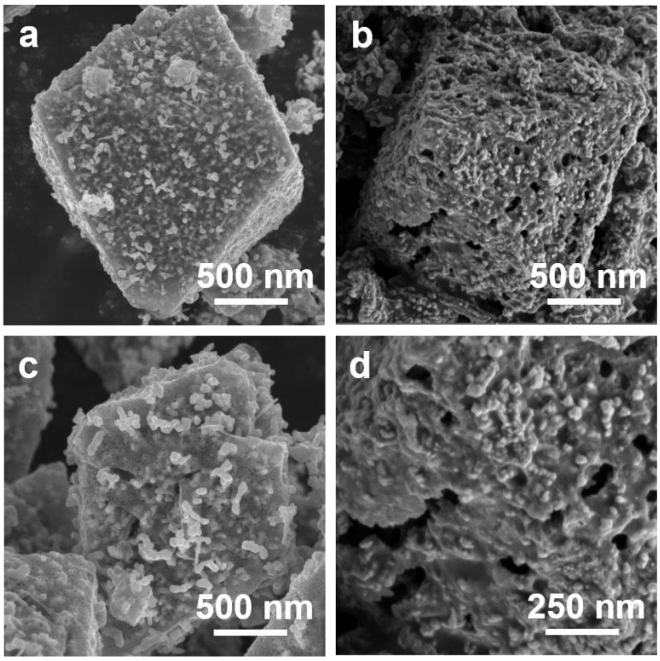

Figure S5. SEM images of the a) Cu@CNHO, b) Cu_3P/Cu@CNHO, and c) Cu_3P@CNHO, respectively. d) The enlarged SEM image of Cu_3P/Cu@CNHO.

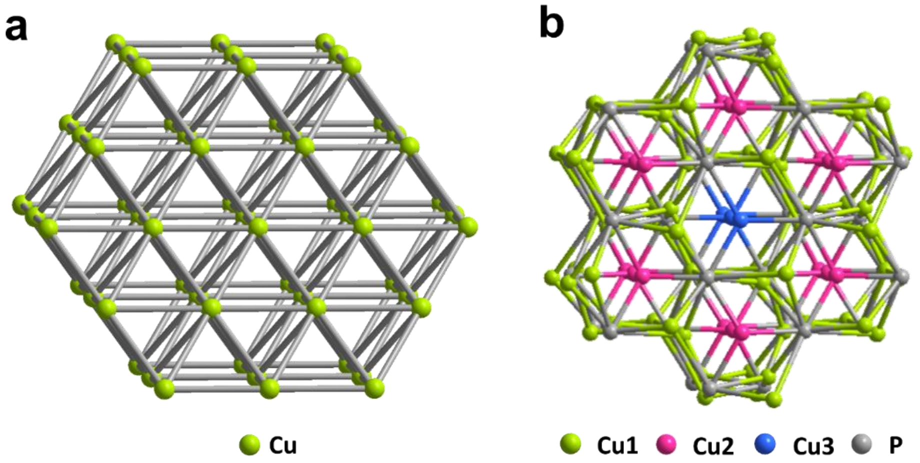

Figure S6. The crystal structure of (a) Cu, (b) Cu_3P. In terms of Cu depicted in Figure S6a, each copper metal atom furnishes a coordination number of twelve, presenting the unique face-centered cubic stack geometrical structure. As for the asymmetric unit of Cu_3P (Figure S6b), there are three crystallographically independent Cu ions. Cu1 is coordinated by four phosphorus atoms in a tetrahedral coordination geometry, Cu2 is coordinated by three phosphorus atoms in a square pyramidal geometry configuration, whereas Cu3 is coordinated by three phosphorus atoms in a trigonal-planar coordination geometry.

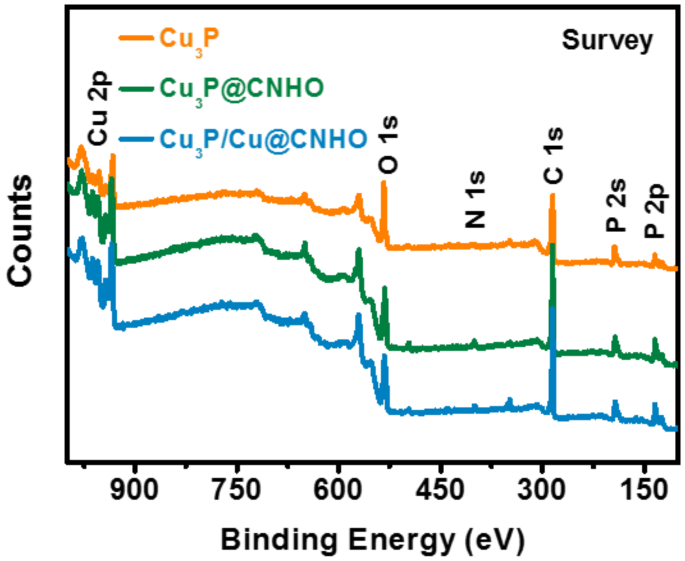

Figure S7. XPS survey spectra of the Cu_3P, Cu_3P@CNHO, and Cu_3P/Cu@CNHO, respectively.

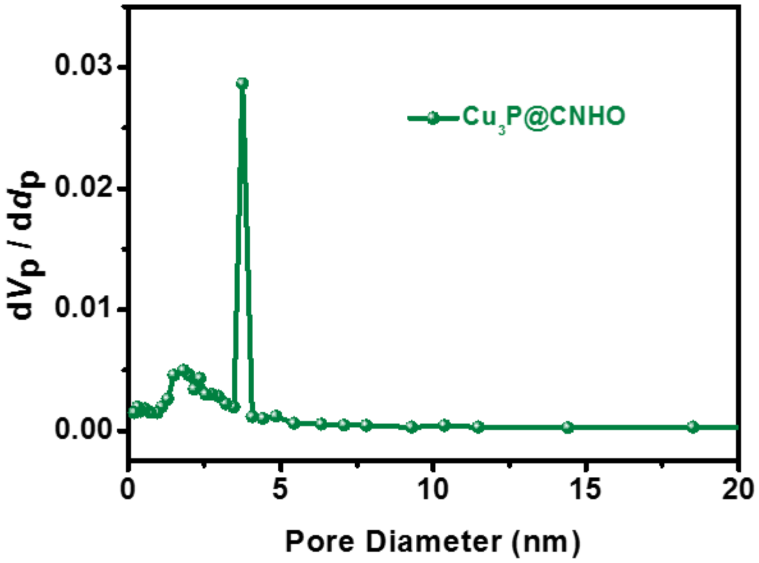

Figure S8. Pore-size distribution curves of the Cu_3P@CNHO.

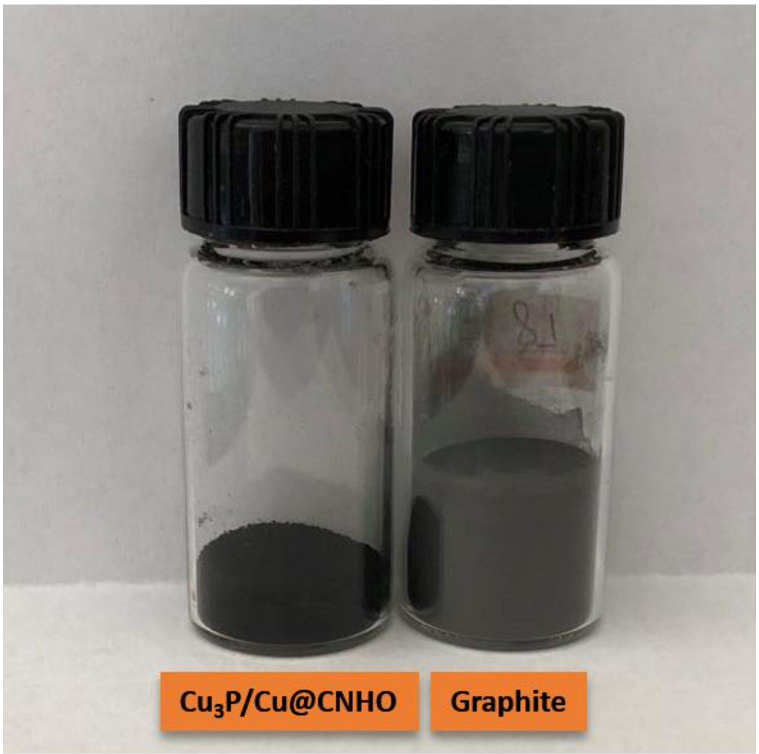

Figure S9. Volume comparison between Cu_3P/Cu@CNHO and commercial graphite with the same mass.

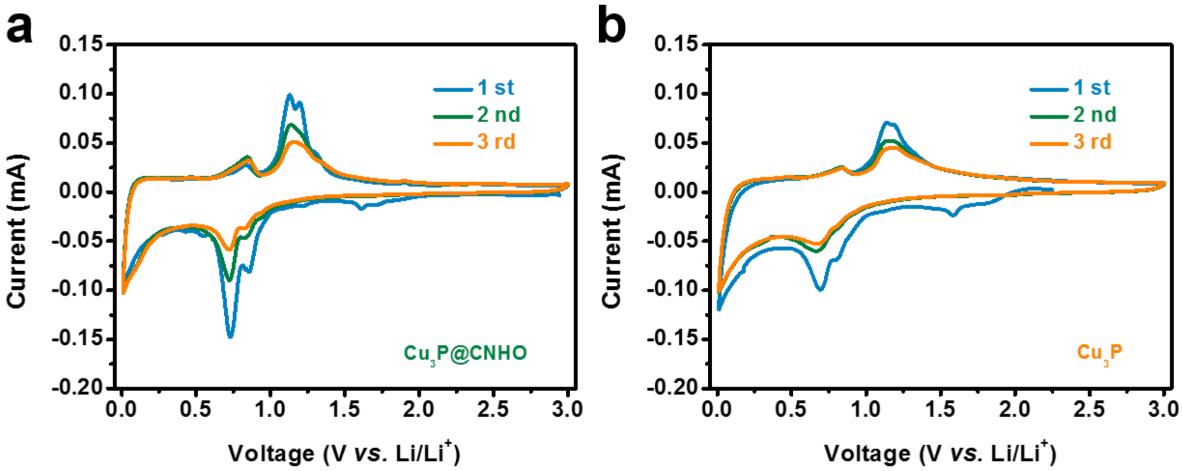

Figure S10. CV curves of the Cu_3P@CNHO and Cu_3P electrodes at a scan rate of 0.2 mV s^{-1} with a voltage window from 0.01 to 3.0 V.

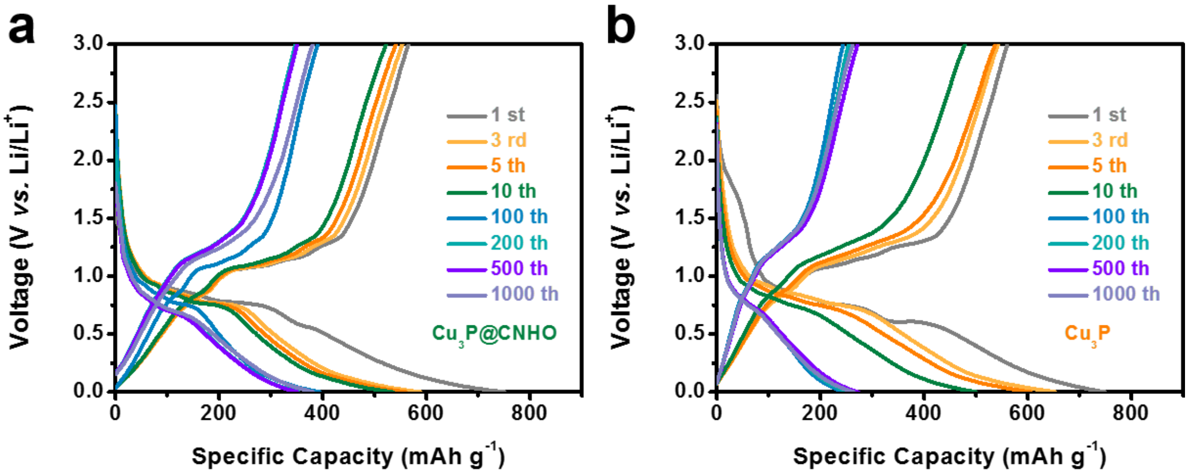

Figure S11. GCD profiles of Cu_3P@CNHO and Cu_3P electrodes at 1 A g^{-1}, respectively.

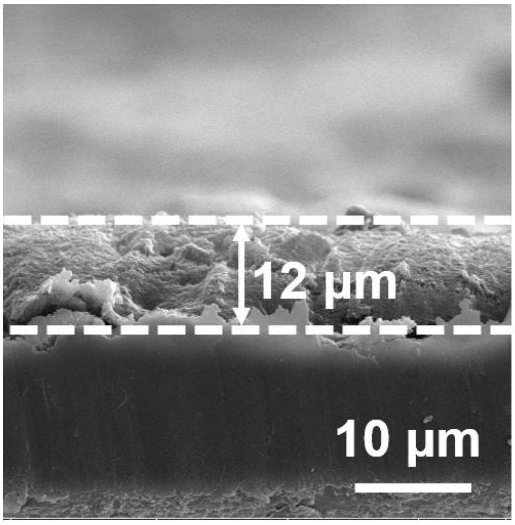

Figure S12. Cross-sectional SEM image of Cu_3P/Cu@CNHO electrode film with a mass loading of 4.9 mg cm^{-2}.

Calculation of the volumetric capacity:

The volumetric capacity (C_v) of Cu_3P/Cu@CNHO electrode is calculated based on the formula: $C_v = C_g * \rho$, where C_g represents for the gravimetric capacity, and ρ equals to the mass density of loading active material for the electrode film. ρ is determined by the equation: $\rho = m_{areal} / T$, where T is the thickness of the electrode film evaluated by the cross-sectional SEM image and m_{areal} is the areal mass loading of the active material. Herein, as for the

Cu_3P/Cu@CNHO electrode film, the values of C_g, m_{areal}, and T are 463.2 mAh g^{-1} at 1 A g^{-1}, 4.9 mg cm^{-2}, and 12 μm, respectively. The C_v is calculated to be 1878.4 mAh cm^{-3} by the above-mentioned means.

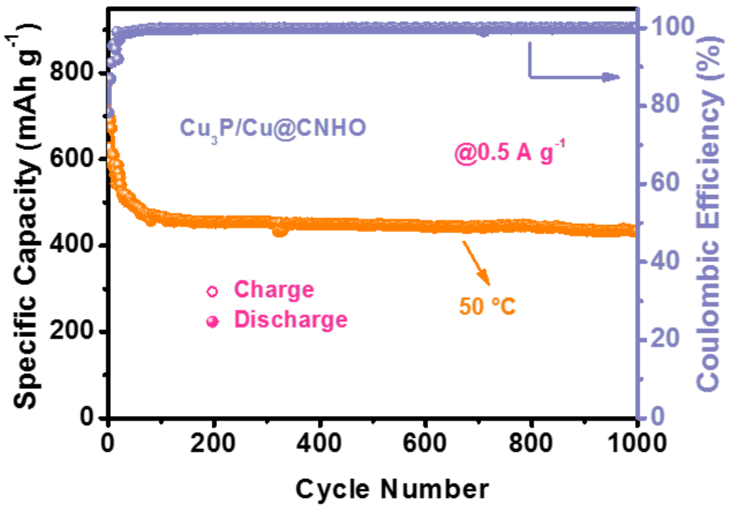

Figure S13. Cycling performance of Cu_3P/Cu@CNHO under an evaluated temperature (50 °C) at 0.5 A g^{-1}.

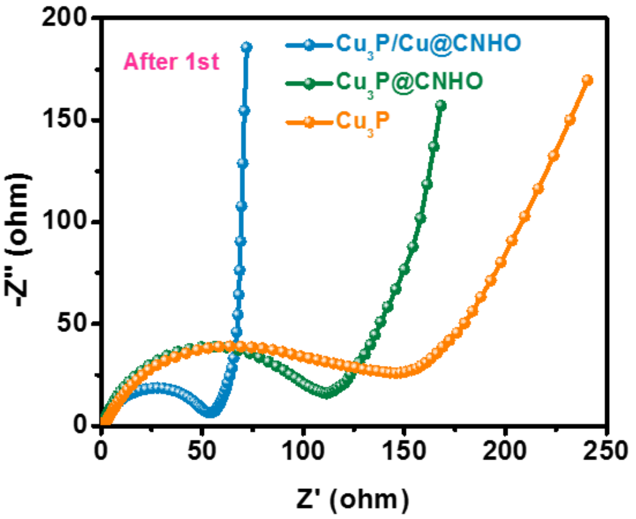

Figure S14. Corresponding Nyquist plots of Cu_3P, Cu_3P@CNHO, and Cu_3P/Cu@CNHO after initial cycle, respectively.

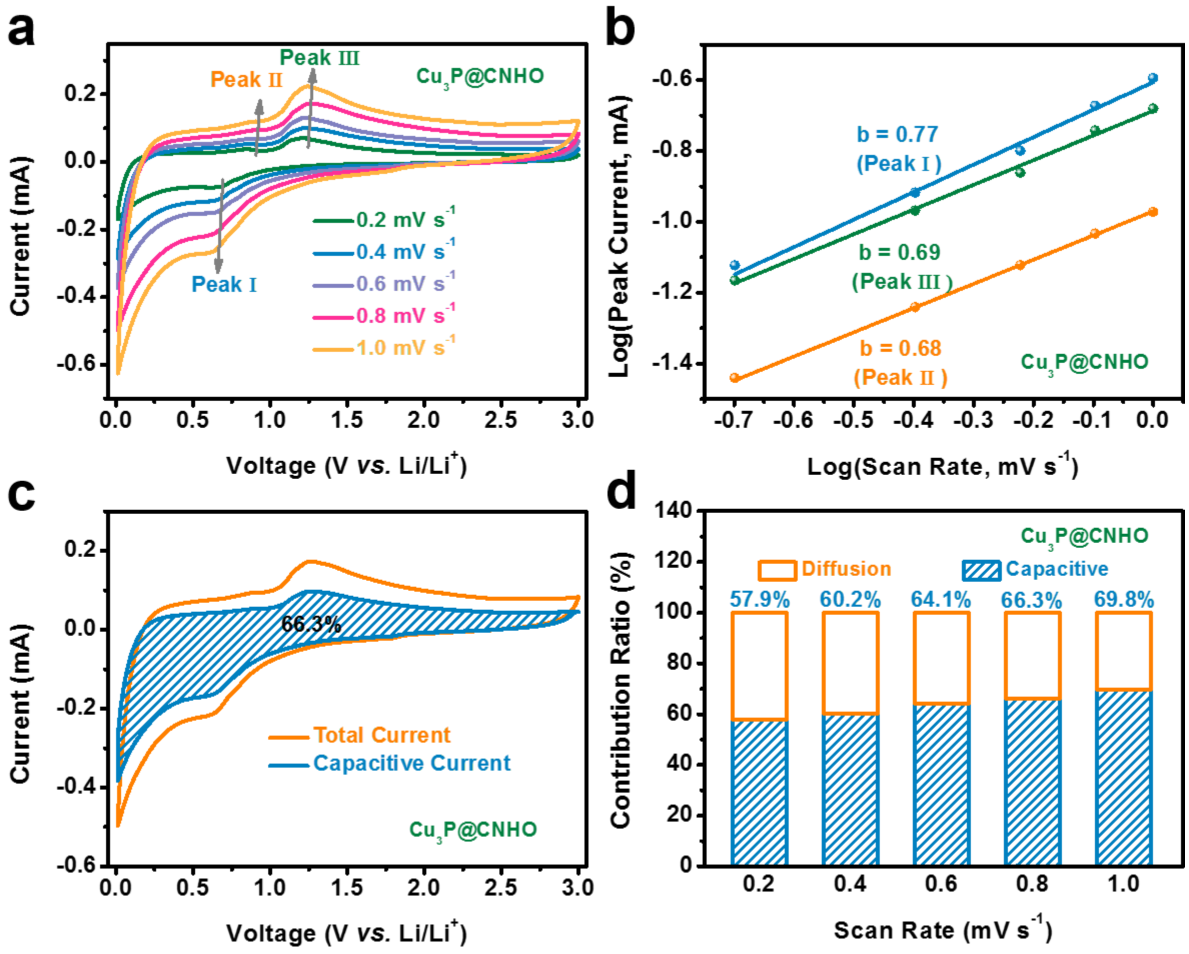

Figure S15. Kinetics analysis of the Li-ion storage performance and quantitative analysis for the pseudocapacitive contribution for Cu_3P@CNHO anode. a) CV plots at increasing sweep rates from 0.2 to 1.0 mV s^{-1}. b) Calculations for the determinational *b* values of main cathodic and anodic peaks. c) Separation of the pseudocapacitive and diffusion-controlled contribution by CV curve at 0.8 mV s^{-1}. d) Contribution percentages of the pseudocapacitive-controlled contributions at corresponding sweep rates.

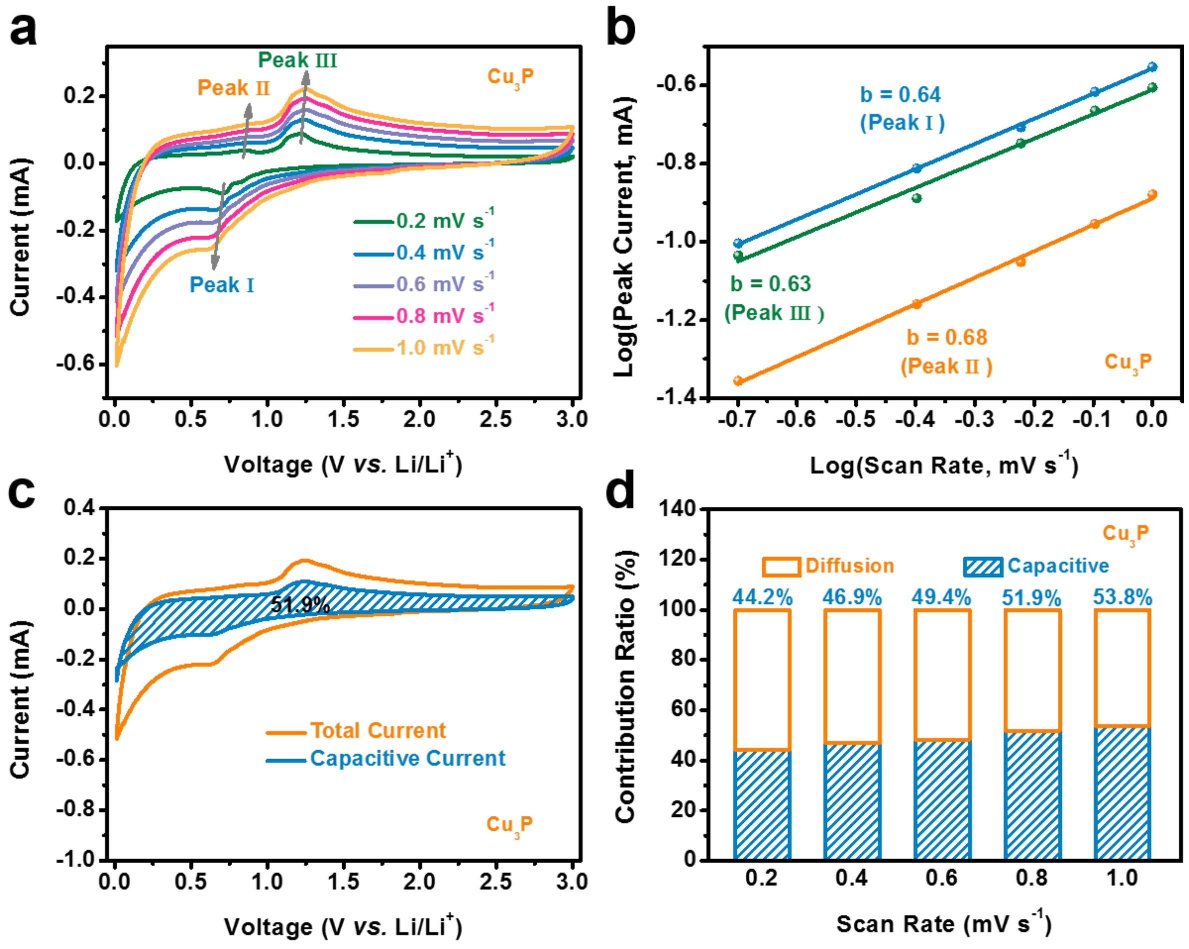

Figure S16. Kinetics analysis of the Li-ion storage performance and quantitative analysis for the pseudocapacitive contribution for Cu_3P anode. a) CV plots at increasing sweep rates from 0.2 to 1.0 mV s^{-1}. b) Calculations for the determinational *b* values of main cathodic and anodic peaks. c) Separation of the pseudocapacitive and diffusion-controlled contribution by CV curve at 0.8 mV s^{-1}. d) Contribution percentages of the pseudocapacitive-controlled contributions at corresponding sweep rates.

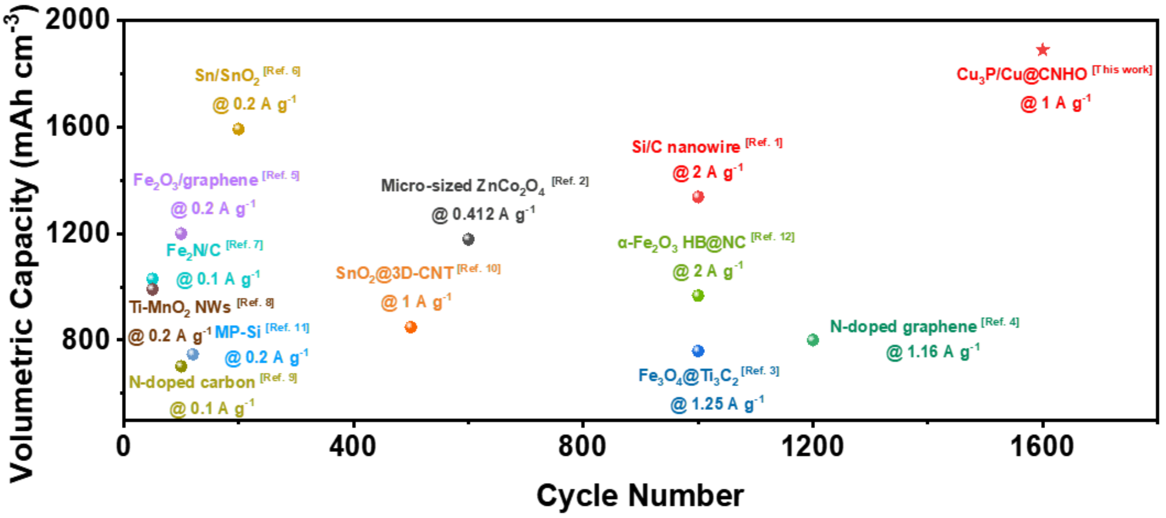

Figure S17. Long-cycling performance comparison between our work and some representative TMO-based and C-based LIB anodes with volumetric capacities.

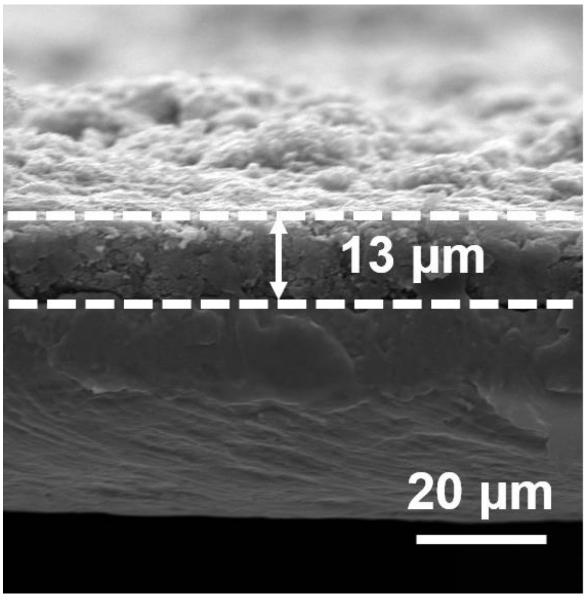

Figure S18. Cross-sectional SEM image of Cu_3P/Cu@CNHO electrode film after cycles.

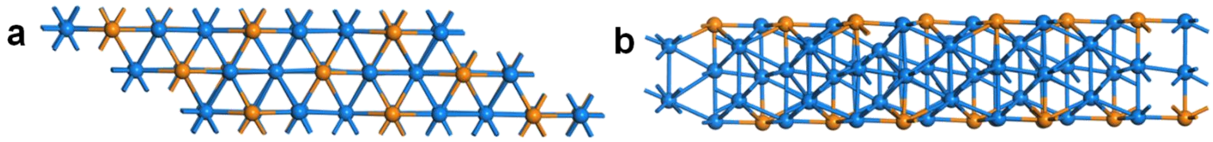

Figure S19. Cluster structure of Cu_3P bulk from the a) top and b) side views. The big blue and orange spheres represent for Cu and P atoms, respectively.

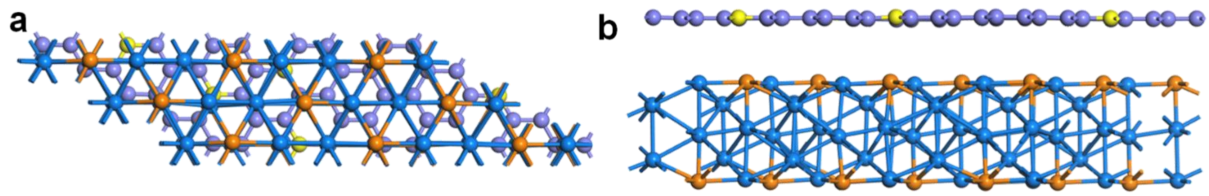

Figure S20. Cluster structure model of the Cu_3P@CNHO heterostructure from the a) top and b) side views. The big blue, orange, purple, and yellow spheres represent for Cu, P, C, and N atoms, respectively.

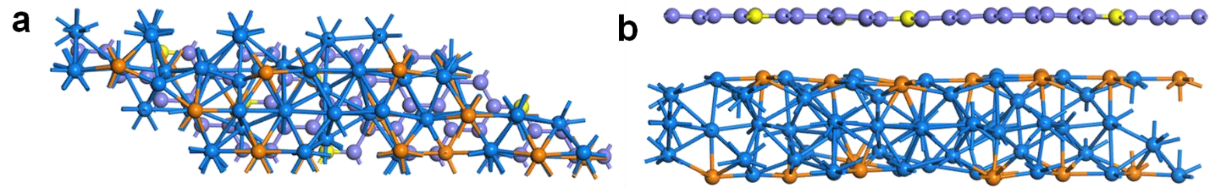

Figure S21. Cluster structure model of the Cu_3P/Cu@CNHO heterostructure from the a) top and b) side views. The big blue, orange, purple, and yellow spheres represent for Cu, P, C, and N atoms, respectively.

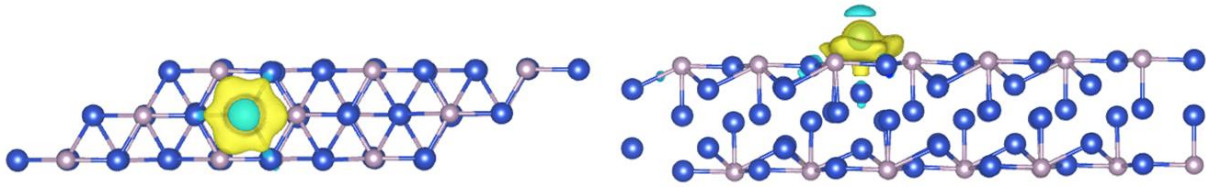

Figure S22. Top and side views of differential charge density distribution of the Cu_3P bulk of Li adsorbing on the outer surface of Cu_3P. Here, the accumulation of electrons is depicted in yellow regions, along with the depletion of electrons is shown in aquamarine regions.

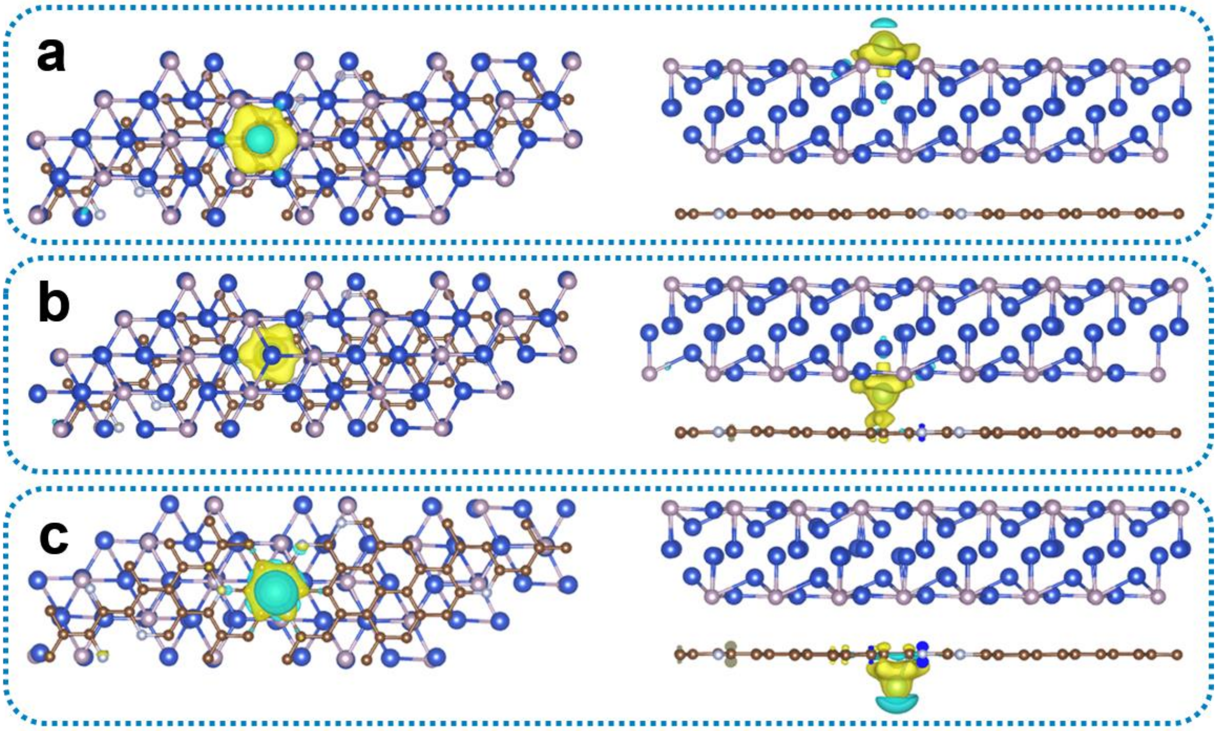

Figure S23. Top and side views of differential charge density distribution of the Cu_3P@CNHO heterostructure of Li: a) adsorbing on the outer surface of Cu_3P; b) inserting into the intralayer of Cu_3P@CNHO; c) adsorbing on the outer surface of CNHO encapsulated-carbon. Here, the accumulation of electrons is depicted in yellow regions, along with the depletion of electrons is shown in aquamarine regions.

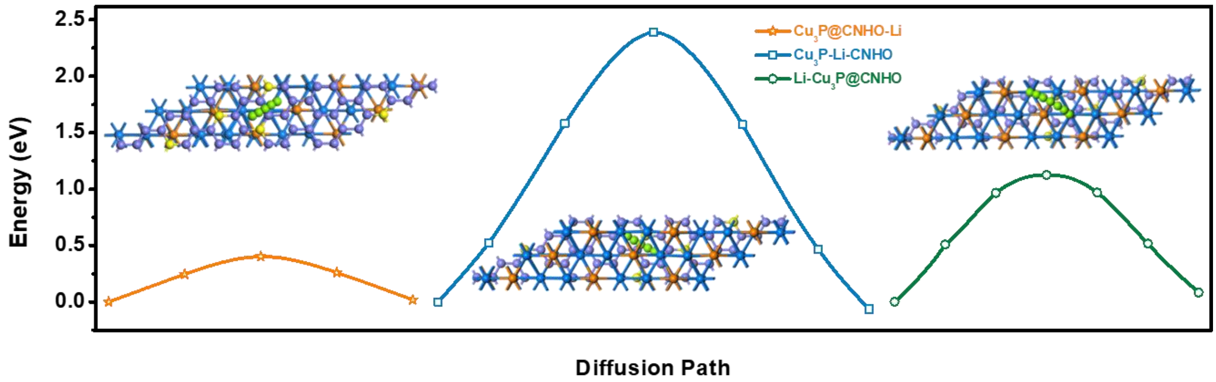

Figure S24. The diffusion pathways and corresponding calculated diffusion energy barrier profiles of Cu_3P@CNHO heterostructure for Li diffusion on I) the outer surface of CNHO encapsulated-carbon (Cu_3P@CNHO-Li), II) the intralayer of Cu_3P@CNHO (Cu_3P-Li-CNHO), and III) the outer surface of Cu_3P (Li-Cu_3P@CNHO). The optimized diffusion paths are indicated by the small green spheres.

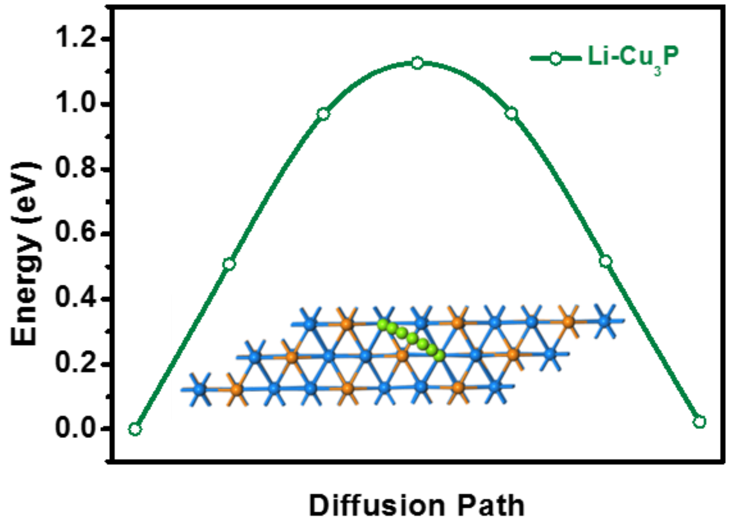

Figure S25. The diffusion pathways and corresponding calculated diffusion energy barrier profiles of Cu_3P for Li migration on outer surface of Cu_3P. The optimized diffusion paths are indicated by the small green spheres.

Table S1. Comparison of the rate capability and long-cycling performance of the Cu_3P/Cu@CNHO electrode with previously reported Cu_3P materials for LIBs.

Samples	Rate capacity		Cycle performance			Loading Density (mg cm^{-2})	Ref.
	capacity retention (%)	Current density (A g^{-1})	Capacity (mAh g^{-1})	Current density (A g^{-1})	Cycle number		
**MOF-derived Cu_3P/Cu@CNHO**	**76.0**	**10**	**463.2**	**1**	**1600**	**4.9**	**This Work**
Cu foam derived Cu_3P/Cu	64.1	2.14	377	0.032	70	0.91	[S13]
Cu foam derived Cu_3P@C-Cu	~40.0	2.5	418	0.15	500	0.7	[S14]
Cu-atom derived Fe-added Cu_3P	155	0.36	178.5	0.18	50	-	[S15]
Cu membrane derived Cu_3P	-	-	250	0.075	20	10.5	[S16]
Cu powder derived Cu_3P	~68.6	0.605	220	0.024	50	-	[S17]
Cu_3P hierarchical dendrites	-	-	291	0.037	20	-	[S18]

References:

[S1] G. Hou, B. Cheng, Y. Yang, Y. Du, Y. Zhang, B. Li, J. He, Y. Zhou, D. Yi, N. Zhao, Y. Bando, D. Golberg, J. Yao, X. Wang, F. Yuan, *ACS Nano* **2019**, *13*, 10179.

[S2] J. Liu, Y. Xuan, D. G. D. Galpaya, Y. Gu, Z. Lin, S. Zhang, C. Yan, S. Feng, L. Wang, *J. Mater. Chem. A* **2018**, *6*, 19455.

[S3] Y. Wang, Y. Li, Z. Qiu, X. Wu, P. Zhou, T. Zhou, J. Zhao, Z. Miao, J. Zhou, S. Zhuo, *J. Mater. Chem. A* **2018**, *6*, 11189.

[S4] X. Wang, L. Lv, Z. Cheng, J. Gao, L. Dong, C. Hu, L. Qu, *Adv. Energy Mater.* **2016**, *6*, 1502100.

[S5] Z. Li, D. Kong, G. Zhou, S. Wu, W. Lv, C. Luo, J.-J. Shao, B. Li, F. Kang, Q.-H. Yang, *Energy Storage Mater.* **2017**, *6*, 98.

[S6] L. Zhang, K. Zhao, C. Sun, R. Yu, Z. Zhuang, J. Li, W. Xu, C. Wang, W. Xu, L. Mai, *Energy Storage Mater.* **2019**, *25*, 376.

[S7] Y. Dong, B. Wang, K. Zhao, Y. Yu, X. Wang, L. Mai, S. Jin, *Nano Lett.* **2017**, *17*, 5740.

[S8] K. Zhao, C. Sun, Y. Yu, Y. Dong, C. Zhang, C. Wang, P. M. Voyles, L. Mai, X. Wang, *ACS Appl. Mater. Interfaces* **2018**, *10*, 44376.

[S9] J. Jin, Z. Wang, R. Wang, J. Wang, Z. Huang, Y. Ma, H. Li, S. H. Wei, X. Huang, J. Yan, S. Li, W. Huang, *Adv. Funct. Mater.* **2019**, *29*, 1807441.

[S10] P. Bhattacharya, J. H. Lee, K. K. Kar, H. S. Park, *Chem. Eng. J.* **2019**, *369*, 422.

[S11] T. Mu, B. Shen, S. Lou, Z. Zhang, Y. Ren, X. Zhou, P. Zuo, C. Du, Y. Ma, H. Huo, G. Yin, *Chem. Eng. J.* **2019**, *375*, 121923.

[S12] M. Qin, Z. Zhang, Y. Zhao, L. Liu, B. Jia, K. Han, H. Wu, Y. Liu, L. Wang, X. Min, K. Xi, C. Y. Lao, W. Wang, X. Qu, R. V. Kumar, *Adv. Funct. Mater.* **2019**, *29,* 1902822.

[S13] S. Ni, J. Ma, X. Lv, X. Yang, L. Zhang, *J. Mater. Chem. A* **2014**, *2*, 20506.

[S14] S. Ni, B. Zheng, J. Liu, D. Chao, X. Yang, Z. Shen, J. Zhao, *J. Mater. Chem. A* **2018**, *6*, 18821.

[S15] A. Zhou, B. Yang, W. Wang, X. Dai, M. Zhao, J. Xue, M. Han, C. Fan, J. Li, *RSC Adv.* **2016**, *6*, 26800.

[S16] M. C. Stan, R. Klöpsch, A. Bhaskar, J. Li, S. Passerini, M. Winter, *Adv. Energy Mater.* **2013**, *3*, 231.

[S17] S. Liu, S. Li, J. Wang, Q. Shi, M. Li, *Mater. Res. Bull.* **2012**, *47*, 3352.

[S18] V. Yarmiayev, Y. Miroshnikov, G. Gershinsky, V. Shokhen, D. Zitoun, *Electrochim. Acta* **2018**, *292*, 846.
